# Sex-based differences in conjunctival goblet cell responses to pro-inflammatory and pro-resolving mediators

**DOI:** 10.1038/s41598-022-20177-9

**Published:** 2022-09-29

**Authors:** Menglu Yang, Haakon K. Fjærvoll, Ketil A. Fjærvoll, Nicholas H. Wang, Tor P. Utheim, Charles N. Serhan, Darlene A. Dartt

**Affiliations:** 1 Department of Ophthalmology, Harvard Medical School, Massachusetts Eye and Ear, Schepens Eye Research Institute, 20 Staniford Street, Boston, MA 02114 USA; 2 Institute of Clinical Medicine, Faculty of Medicine, University of Oslo, Oslo, Norway; 3 Department of Medical Biochemistry, Oslo University Hospital, Oslo, Norway; 4 Department of Ophthalmology, Oslo University Hospital, Oslo, Norway; 5 Department of Anesthesia, Center for Experimental Therapeutics and Reperfusion Injury, Brigham and Women’s Hospital and Harvard Medical School, Boston, MA USA

**Keywords:** Chronic inflammation, Calcium signalling

## Abstract

Many conjunctival inflammatory diseases differ between the sexes and altered conjunctival goblet cells (CGCs) response is often involved. Inflammation is initiated by the release of pro-inflammatory mediators and terminated by the biosynthesis of specialized pro-resolution mediators (SPMs). Herein, we determined the sex-based difference in the responses of CGCs to inflammatory stimuli or pro-resolving lipid SPMs and their interaction with sex hormones. GCs were cultured from pieces of human conjunctiva in RPMI media. CGCs were transferred 24 h before the start of experiments to phenol red-free and FBS-free media to minimize exogenous hormones. RT-PCR, immunofluorescence microscopy (IF), and Western Blot (WB) were performed to determine the presence of sex hormone receptors. Cellular response to pro-inflammatory stimuli or SPMs was studied by measuring the increase in intracellular [Ca^2+^] ([Ca^2+^]_i_) using fura 2/AM microscopy. Use of RT-PCR demonstrated estrogen receptor (ER) α in 4/5 males and 3/3 females; ERβ in 2/4 males and 2/3 females; and androgen receptors (AR) in 3/3 male and 3/3 female CGCs. Positive immunoreactivity by IF and protein expression by WB was detected using antibodies for the ERα and ERβ in 3/3 males and 3/3 females, while AR were only present in males. Significantly different Ca^2+^ responses between sexes were found with carbachol only at 10^–3^ M, but not with histamine or leukotriene (LT) B_4_ at any concentration used. Incubation with dihydrotestosterone (DHT), estrone (E1), or estradiol (E2) at 10^–7^ M for 30 min significantly inhibited the LTB_4_-stimulated [Ca^2+^]_i_ increase in male and female CGCs. Incubation with DHT, E1, and E2 overnight significantly inhibited the LTB_4_ response in females, while DHT and E2 significantly inhibited the LTB_4_ response in males. The SPM lipoxin A_4_ (LXA_4_) (10^–9^–10^−8^ M), but not the resolvins D1 or D2, induced an [Ca^2+^]_i_ increase that was significantly higher in males compared to females. We conclude that male and female CGCs showed differences in the expression of sex hormone receptors. Treatment with sex hormones altered pro-inflammatory mediator LTB_4_-induced response. Males compared to females have a higher response to the ω-6-fatty acid derived SPM LXA_4_, indicating males may terminate inflammation in conjunctival goblet cells faster than females.

## Introduction

The prevalence of many ocular surface diseases differs between men and women. In the US, women have an age-adjusted 70% increase compared to men in the risk of dry eye disease^1,2^, and more antihistamine medicines were prescribed to women^3^. The differences in biological characteristics are one of the major probable causes of this phenomenon^3^. Sex affects health at multiple biological levels, from chromosomes to sex hormones. In leukocytes, X and Y chromosomes have different profiles of immune system-related genes^4^; while sex hormones also moderate immunity. For example, estrogens at the level of the ovulatory phase or pregnancy suppress cytotoxicity of natural killer (NK) cells, interferon-γ production, and T-cell subpopulation and their effect on the innate immune response^5^. Several autoimmune diseases such as systemic lupus erythematosus (SLE), Sjogren’s disease (SJD), Grave’s disease (GD), rosacea, and rheumatoid arthritis (RA) have a higher prevalence in women than men^6^. These findings suggest that sex affects the immune and inflammatory processes, and that sex-based differences are present at both cellular and systemic levels.

In the eye, sex-based differences are observed in multiple components of the eye^7–11^ and impact multiple processes^12–14^. Sex hormones modulate tear film stability and homeostasis of the ocular surface^15^. Sex-based differences were also demonstrated in the resolution of inflammation in dry eye by an action in females to decrease regulation of the immune response of the ocular surface by the pro-resolving mediator lipoxin(LX) A_4_ that exacerbates dry eye^13^. Disturbances of the tear film are often found in patients with alteration of hormone levels such as menopause or pregnancy^15^. Furthermore, androgens stimulate meibomian gland secretion by transcriptome regulation, while estrogens induce proinflammatory gene expression in corneal epithelial cells and meibomian glands^16^. In the conjunctiva, the number of goblet cells is higher in men than women, and in women, the goblet cell count decreases around ovulation^17^, suggesting additional sex differences in goblet cells. However, little additional work has been published on the sex-based differences in the conjunctiva and its goblet cells, a main component of the ocular surface.

The conjunctiva surrounds the cornea, overspreads the sclera, and lines the eyelids. The conjunctival goblet cell, a major cell type of the conjunctival epithelium, synthesizes, stores, and secretes MUC5AC, a large, secretory gel-forming mucin. This mucin forms the inner layer of the tear film and protects the ocular surface from exogenous agents. Physiologically conjunctival goblet cell secretion is induced by stimulating a complex reflex arc consisting of the afferent sensory nerves in the cornea and conjunctiva that activate efferent parasympathetic and sympathetic nerves with efferent nerve endings surrounding the goblet cells^18^. The mucin secretion function is closely mediated by the intracellular [Ca^2+^] ([Ca^2+^]_i_)^19^.

In ocular surface inflammation, evidence suggests that the goblet cell is one of the targeted cell types^20^. In inflamed eyes, goblet cells can be stimulated by infiltrating pro-inflammatory mediators, including prostaglandins (PGs), leukotrienes (LTs), and allergic mediators such as histamine^21^. The excessive mucin secretion causes catarrhous and discomfort^20,22^, which happens in chronic inflammatory diseases such as allergic conjunctivitis and early dry eye. To actively terminate inflammation and restore homeostasis, specialized pro-resolving mediators (SPMs) are biosynthesized and released^23^. There are multiple families of SPMs identified, including LXs, resolvins (Rv), maresins (Mar), and protectins, biosynthesized from omega 6 and omega 3 fatty acids. Members of the D- and E series resolvins, as well as lipoxins and maresins can stimulate conjunctival goblet cell secretion to maintain ocular surface homeostasis and terminate the excessive secretion induced by proinflammatory mediators^24–27^. Furthermore, multiple SPMs are present in human emotional tears with different concentrations in men and women^17^.

Herein, we studied the presence of the sex hormone receptors and their regulatory action on normal function of conjunctival goblet cells (CGC). We also investigated the sex-based differences in cellular response of goblet cells in homeostasis by using a cholinergic agonist (carbachol, Cch); in acute inflammation by using LTB_4_; in allergy by using histamine, and in resolution of inflammation by treating with two of the D-series resolvins and LXA_4_. We hypothesize that conjunctival goblet cells respond differently to pro-inflammatory and pro-resolving stimuli in men than women and that the goblet cell response can be altered by exogenous sex hormones.

## Results

### Differential expression of sex hormone receptors in goblet cells cultured from male compared to female human conjunctiva

**Figure 1:**
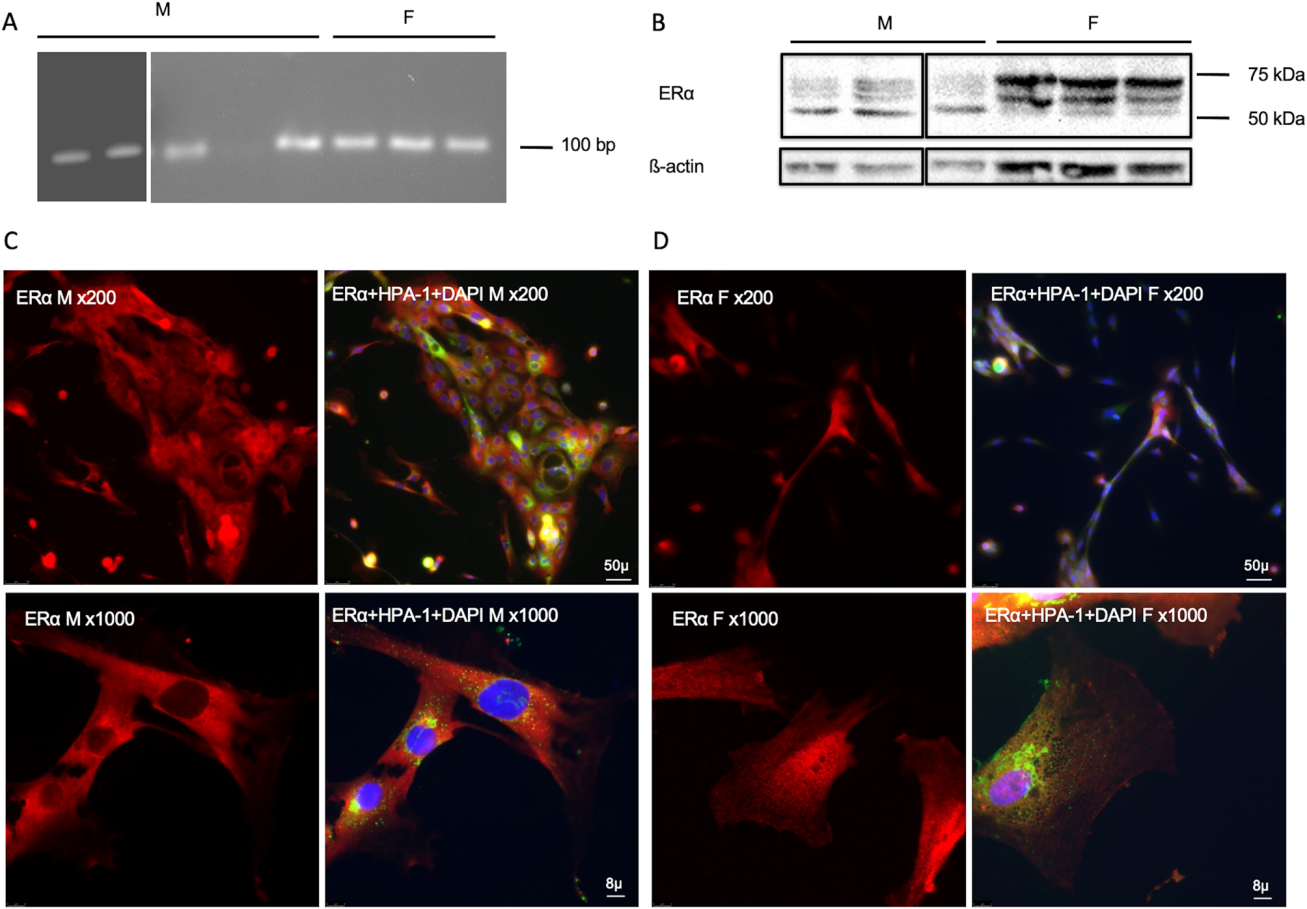
Identification of ERα in human conjunctival goblet cells cultured from males and females. RNA was isolated from cultured goblet cells and RT-PCR performed using primers for human *Erα*. A single band was detected at around 100 bp in samples from 4 out of 5 male (M) and 3 out of 3 females (F) (**A**). Protein samples were collected from cell pellets and Western Blot analysis was performed using antibody against ERα. A major band at 50 kDa was detected in males, while a major band at 75 kDa and a minor band between 75 and 50 kDa were detected in females (**B**). Immunofluorescence microscopy was performed on cultured goblet cells using antibodies to ERα (**C** and **D**). (**C**) indicate immunofluorescence to ERα (red) in male cells; (**D**) indicate immunofluorescence to ERα (red) in female cells; an overlay of anti-ERα, HPA-1 (green, indicates the secretory granules of goblet cells), and DAPI (blue, indicates cell nuclei) is shown next to the single channel image of ERα. Magnification was × 200 in the upper panels; × 1000 in the lower panels. Full gels and blots are included in “Supplementary information”. Original blots/gels are presented in Supplementary Figs. 5 and 6.

To determine the expression of the estrogen and androgen receptors in conjunctival goblet cells, RT-PCR and WB were performed using cultured human goblet cells. IF was used to determine the cellular location of the sex hormone receptors on conjunctival goblet cells grown on coverslips. *Erα* mRNA was expressed in 4 out of 5 males and 3 out of 3 females at the expected size of 100 bp (Fig. 1A). ERα protein was present in 3 out of 3 males and 3 out of 3 females by WB analysis (Fig. 1B). Helix pomatia lectin (HPA), a marker of goblet cell secretory product, was present in both male and female goblet cells as a rough granular pattern indicating secretory granules, consistent with previous publications^27^. ERα immunoreactivity was present as a fine punctate pattern concentrated in the cytoplasm in males (Fig. 1C) and the same punctate pattern in both the cytoplasm and nucleus females (Fig. 1D).

**Figure 2:**
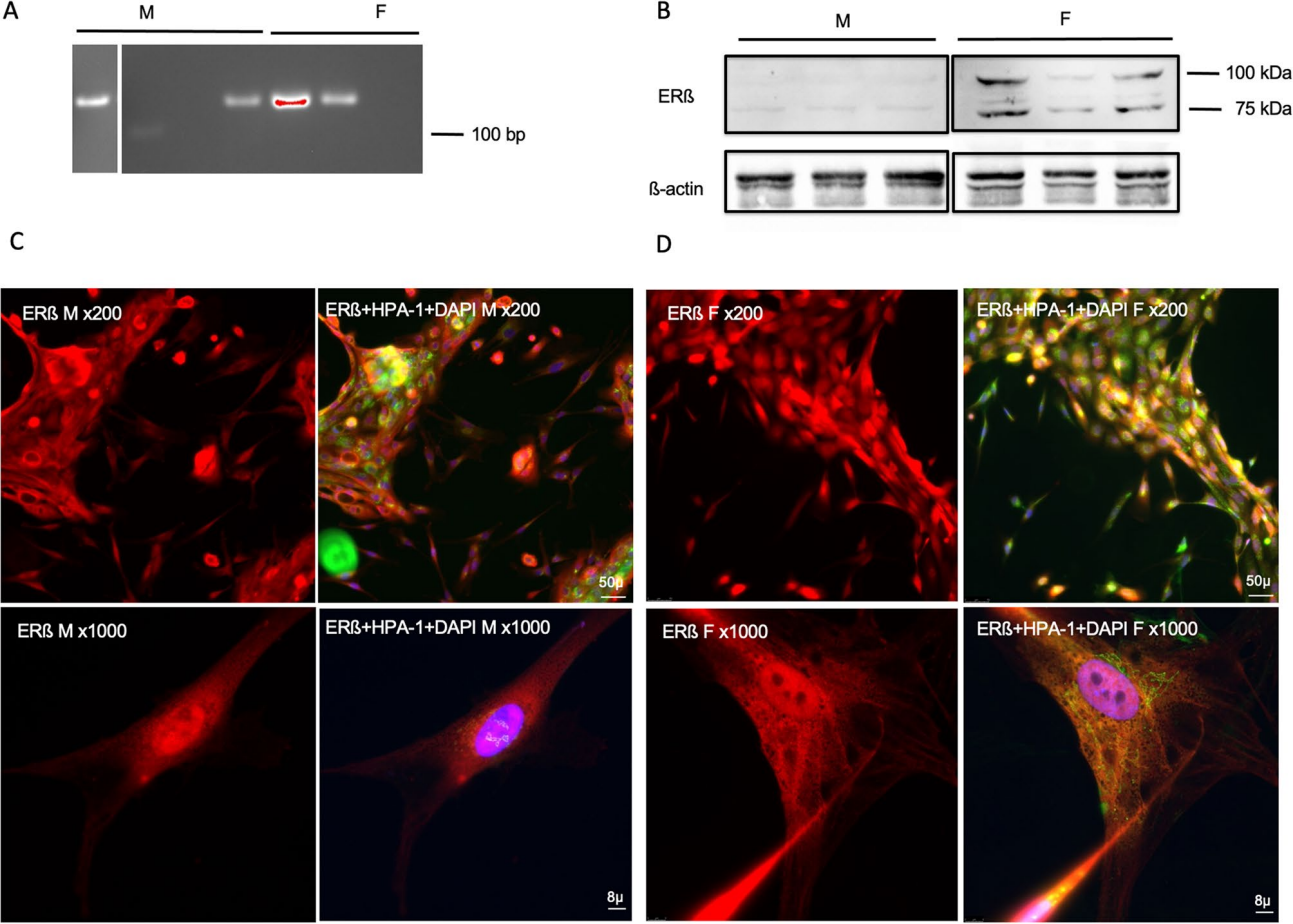
Identification of ERβ in human conjunctival goblet cells cultured from males and females. RNA was isolated from cultured goblet cells and RT-PCR performed using primers for human *Erβ*. A single band was detected at around 100 bp in 2 out of 4 samples from males (M) and 2 out of 3 samples from females (F) (**A**). Protein samples were collected from cell pellets and Western Blot analysis was performed using antibody against ERβ. Two bands at 100 and 75 kDa respectively were detected in female samples (**B**). Trace immunoreactivity of the same size as in females was present in male samples (**B**). Immunofluorescence microscopy was performed on cultured goblet cells using antibodies to ERβ (**C** and **D**). (**C**) indicate immunofluorescence to ERβ (red) in male cells; (**D**) indicate immunofluorescence to ERα (red) in female cells; an overlay of anti-ERβ, HPA-1 (green, indicates the secretory granules of goblet cells), and DAPI (blue, indicates cell nuclei) is shown next to the single channel image of ERβ. Magnification was × 200 in the upper panels; × 1000 in the lower panels. Original blots/gels are presented in Supplementary Figs. 5 and 6.

*Erβ* was found in 2 out of 4 males and 2 out of 3 females at the expected size of 143 bp by RT-PCR (Fig. 2A). In WB, ERβ presented as a double band in 3 out of 3 females, and a trace of ERβ band was found in 3 out of 3 male samples (Fig. 2B). ERβ immunoreactivity was detected as a punctate pattern throughout the cytoplasm and the nuclear area in both males (Fig. 2C) and females (Fig. 2D).

**Figure 3:**
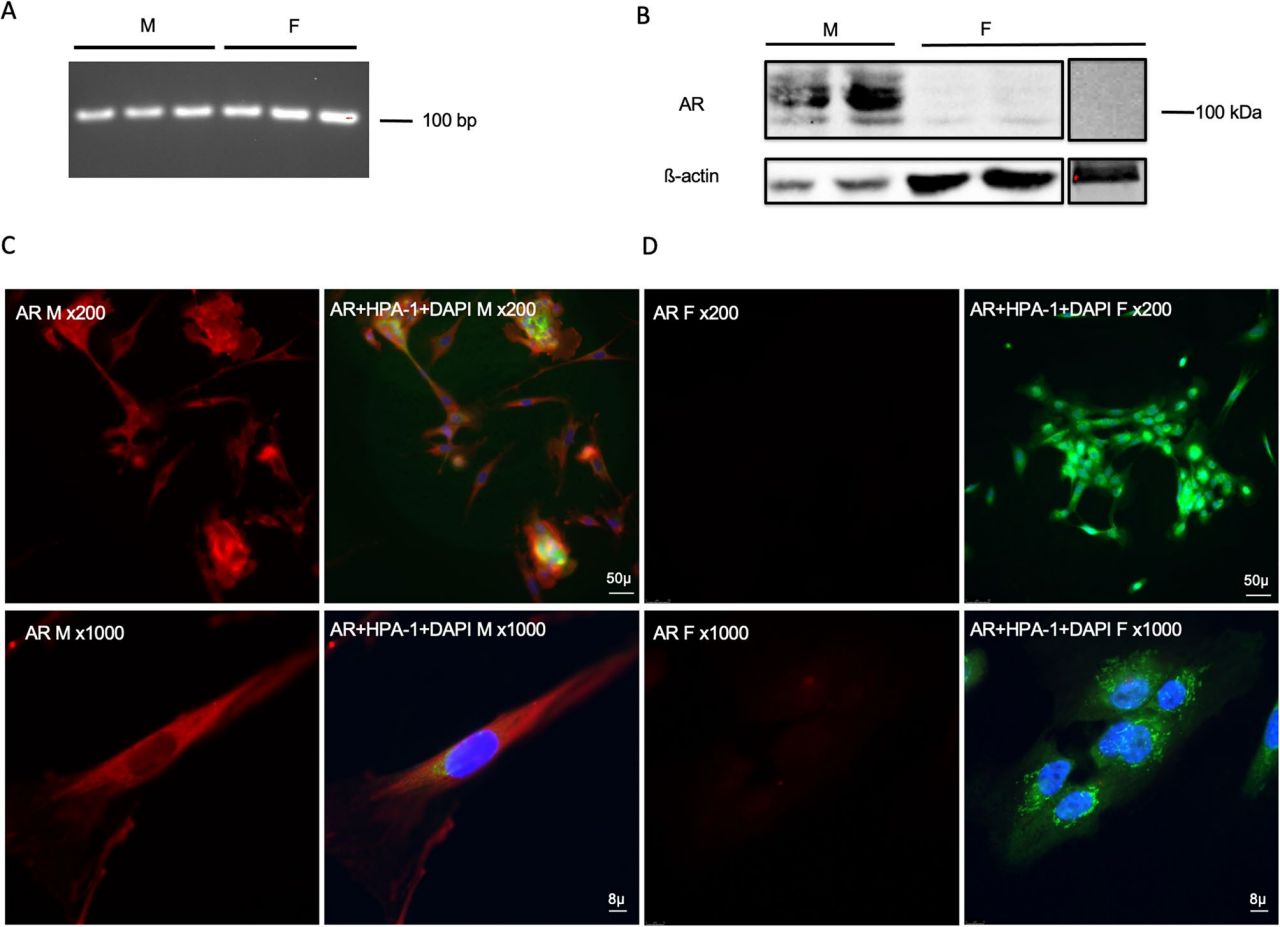
Identification of AR in human conjunctival goblet cells cultured from males and females. RNA was isolated from cultured goblet cells and RT-PCR performed using primers for human *Ar*. A single band was detected at around 100 bp in samples from both males (M) and females (F) (**A**). Protein samples were collected from cell pellets and Western Blot analysis was performed using antibody against AR. A major band at 100 kDa was detected in males while no immunoreactivity was detected in females (**B**). Immunofluorescence microscopy was performed on cultured goblet cells using antibodies to AR (**C** and **D**). (**C**) Indicate immunofluorescence to AR (red) in male cells; (**D**) indicate immunofluorescence to AR (red) in female cells; an overlay of anti-AR, HPA-1 (green, indicates the secretory granules of goblet cells), and DAPI (blue, indicates cell nuclei) is shown next to the single channel image of AR. Magnification was × 200 in the upper panels; × 1000 in the lower panels. Original blots/gels are presented in Supplementary Figs. 5 and 6.

*Ar* mRNA was identified in 3 out of 3 males and 3 out of 3 females at the expected size of 106 bp (Fig. 3A). AR was detected by WB in 2 out of 2 male samples, however, no protein was detected in 3 out of 3 females (Fig. 3B). AR immunoreactivity appeared as a punctate pattern that was more concentrated in the cytoplasm than the nucleus in males (Fig. 3C), but no AR immunoreactivity was detected in females (Fig. 3D). Incubation with the isotype control antibody in IF showed no apparent immunoreactivity for any of the three receptors (Supplemental Fig. 1) indicating that the antibodies used were specific for each receptor.

In summary, the sex hormone receptors are differentially present in human conjunctival GCs from males and females. Although little difference was found in the expression of mRNA for all three receptors, the pattern of expression was very different at the protein level. The ERβ protein was more abundant in females than males. In contrast, AR protein was only found in males. ERα showed the most consistency between the sexes, but a sex-based difference can still be detected in the subcellular localization.

### Differential response of goblet cells cultured from male and female conjunctiva to cholinergic agonists but not allergic mediators

To determine the rapid, short-term activation of conjunctival goblet cells, we used three different types of known goblet cell stimuli: (1) the cholinergic agonist Cch (10^–5^–10^–3^ M) to mimic physiological stimulation by the parasympathomimetic neurotransmitter acetylcholine, (2) LTB_4_ (10^–11^–10^–8^ M) an eicosanoid mediator of allergic conjunctivitis as well as of other types of inflammation and (3) histamine (10^–8^–10^–5^ M) a major mediator of allergic conjunctivitis. The stimuli were applied to male and female goblet cells, and the change in [Ca^2+^]_i_ was measured.

**Figure 4:**
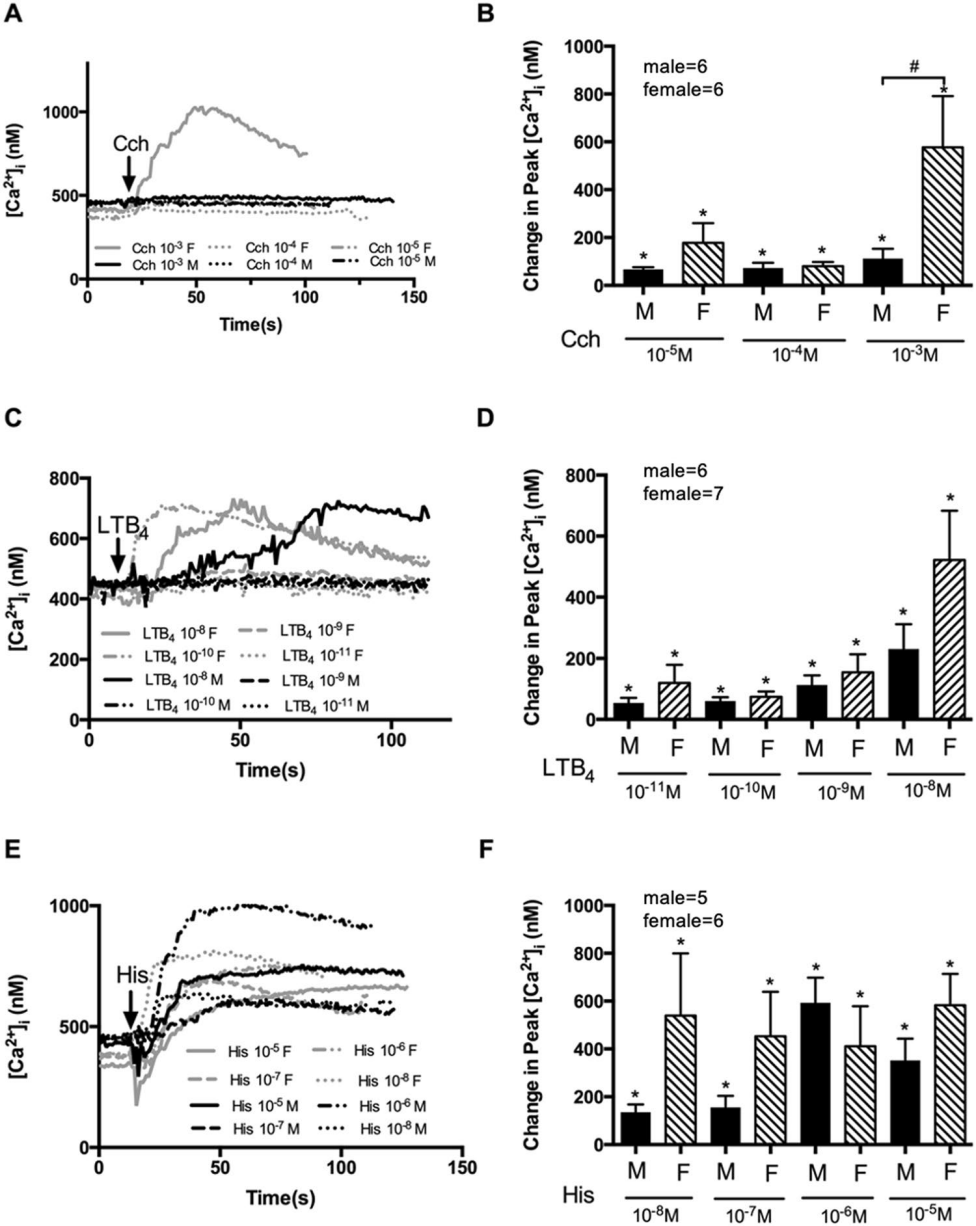
Human conjunctival goblet cells cultured from males and females respond to a neurotransmitter, inflammatory mediator, and allergic mediator. Cultured goblet cells from both sexes were stimulated with carbachol(Cch) 10^–5^–10^−3^ M (**A** and **B**) or LTB_4_ 10^–11^–10^−8^ M (**C** and **D**) or histamine(His) 10^–8^–10^−5^ M (**E** and **F**). [Ca^2+^]_i_ measured by fura2 assay. The average [Ca^2+^]_i_ level over time was shown in (**A**, **C** and **E**); Change in peak [Ca^2+^]_i_ was calculated and shown in (**B**, **D** and **F**). Number of individuals is indicated on each graph. Data are mean ± SEM; the number of individuals is marked in each graph. Arrow indicates the addition of stimuli. Black lines and solid black bars indicate data from male cells, grey lines and slash-patterned bars indicate data from female cells. *Significance difference from basal. ^#^Significance difference between male and female values.

Cch (10^–5^–10^–3^ M) compared to basal significantly increased [Ca^2+^]_i_ in both male and female cells (Fig. 4A,B). Only at a carbachol concentration of 10^–3^ M was the peak [Ca^2+^]_i_ response significantly different between males and females at 112.2 ± 40.54 nM in males and 577.0 ± 213.9 nM in females (*p* < 0.05) (Fig. 4B).

LTB_4_ used at 10^–11^ to 10^–8^ M caused an increase in [Ca^2+^]_i_ in both male and female goblet cells with a maximum response to LTB_4_ at 10^–8^ M with the [Ca^2+^]_i_ significantly increased to 230.8 ± 80.44 nM in males and 521.6 ± 161.6 nM in females (Fig. 4C,D). No significant difference in Ca^2+^ response was found at any concentration of LTB_4_ when comparing the response of male and female cells (Fig. 4C,D).

When the goblet cells were stimulated by histamine, histamine caused an increase in peak [Ca^2+^]_i_ in male but not female goblet cells (Fig. 4E,F). The maximum increase in [Ca^2+^]_i_ of 593.3 ± 104.5 nM occurred at 10^–6^ M histamine in males. In contrast, the maximum increase in [Ca^2+^]_i_ of 583.3 ± 130.1 nM was detected at 10^–5^ M in females (Fig. 4E). No significant difference in peak [Ca^2+^]_i_ was found between the two groups at any histamine concentration (Fig. 4F).

In all, the cellular response of goblet cells to different stimuli that mimic physiological nerve stimulation, inflammation, and allergy was not different between males and females except for the highest concentration of the cholinergic agonist, in which females demonstrated a greater response than males.

### Sex hormones inhibit conjunctival goblet cell response to the eicosanoid inflammatory mediator LTB_4_, but not cholinergic agonist carbachol nor the autacoid histamine

To study the action of sex hormones on cellular responses to inflammatory mediators, we treated goblet cells from male and female conjunctiva with a sex hormone alone or for varying times followed by the addition of LTB_4_. The sex hormones used were dihydrotestosterone (DHT), estrone (E1), and 17-β-estradiol (E2). DHT is a highly potent form of testosterone, estrone a low potency estrogen synthesized in peripheral tissues, and estradiol a high potency estrogen mainly synthesized in the ovary. The addition of 10^–7^ M DHT, E1, or E2 alone did not significantly increase the [Ca^2+^]_i_ compared to basal in conjunctival goblet cells of a mixed series of both sexes (Supplemental Data 2). Histamine at 10^–5^ M, the positive control significantly increases the [Ca^2+^]_i_ from basal.

**Figure 5:**
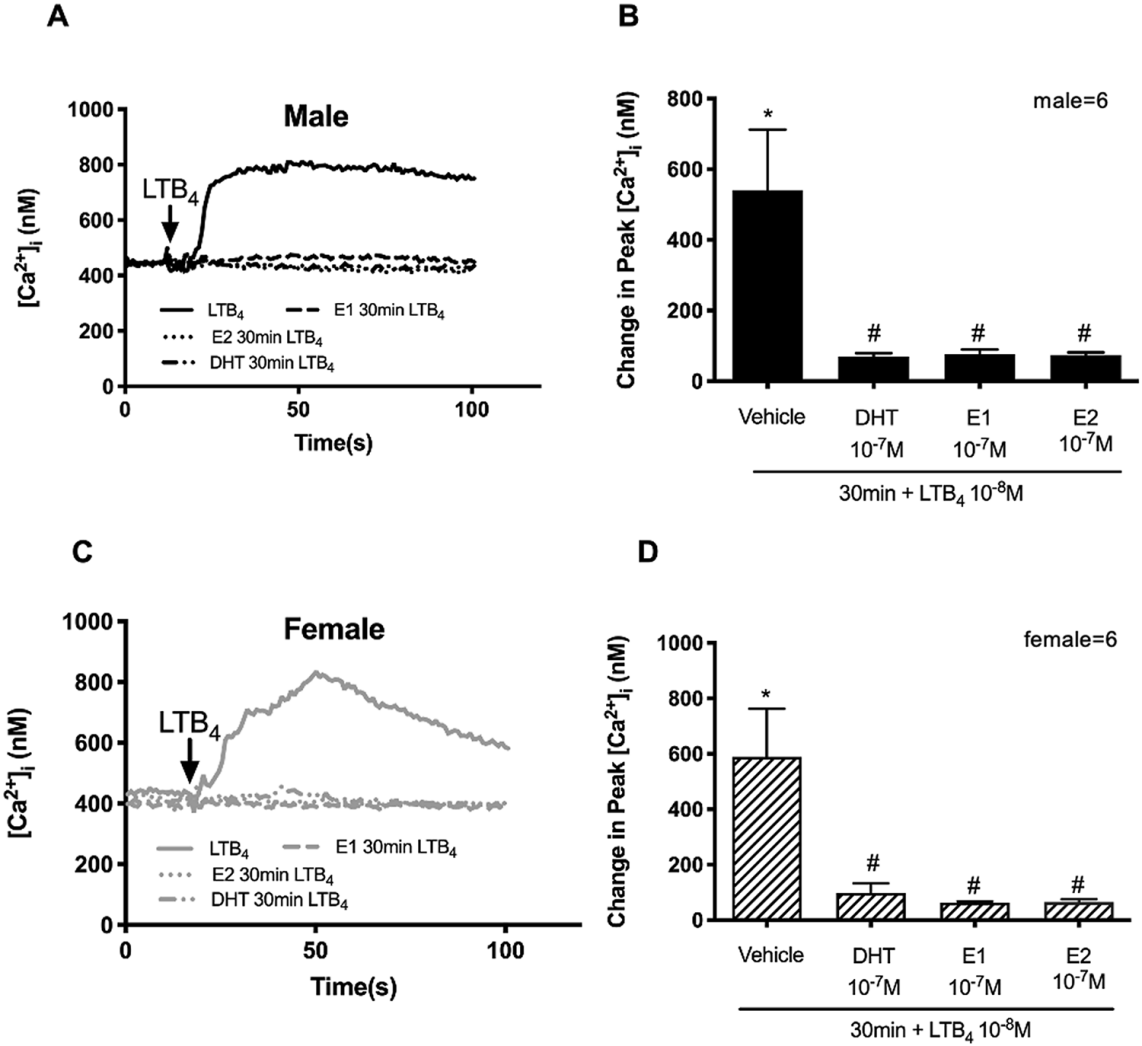
Short-term treatment with sex hormones inhibits the Ca^2+^ response from human conjunctival goblet cells cultured from male and female cells to the inflammatory mediator LTB_4_. Cultured goblet cells from male and female conjunctiva were treated with DHT, E1 or E2 (10^−7^ M) or vehicle for 30 min prior to addition LTB_4_ (10^−8^ M). [Ca^2+^]_i_ was measured by fura2 assay. The average [Ca^2+^]_i_ level over time was shown in (**A** and **C**); Change in peak [Ca^2+^]_i_ was calculated and shown in (**B** and **D**). Data are mean ± SEM, n = 6 for male cells and n = 6 for female cells. Black lines (**A**) and solid bars (**B**) indicate data from male cells. Grey lines and slash-patterned bars indicate data from female cells. *Significance difference from basal. ^#^Significance difference between male and female cells.

To determine the short-term action of sex hormones on physiological, inflammatory, and allergic agonists, 10^–7^ M DHT, E1, or E2 or vehicle were administered to goblet cells 30 min prior to addition of LTB_4_ (10^–8^ M). In male cells, LTB_4_ at 10^–8^ M significantly increased the [Ca^2+^]_i_ to 540.90 ± 171.20 nM (*p* < 0.05) (Fig. 5A,B). A 30 min treatment with DHT significantly decreased the [Ca^2+^]_i_ to 70.02 ± 8.70 nM (*p* < 0.05); with E1 significantly decreased the [Ca^2+^]_i_ to 76.70 ± 13.04 nM (*p* < 0.05); and with E2 significantly decreased the [Ca^2+^]_i_ to 74.32 ± 7.4 nM (*p* = 0.04) (Fig. 5A,B). In female cells, LTB_4_ 10^–8^ M alone significantly increased the [Ca^2+^]_i_ to 559.7 ± 209.2 nM from basal (Fig. 5C,D). A 30 min treatment with DHT significantly decreased the [Ca^2+^]_i_ to 97.60 ± 35.37 nM (*p* < 0.05) (Fig. 5C,D); with E1 significantly decreased the [Ca^2+^]_i_ to 62.95 ± 5.11 nM (*p* < 0.05) (Fig. 5D); and with E2 significantly decreased the [Ca^2+^]_i_ to 65.15 ± 10.58 nM (Fig. 5D).

The short-term action of sex hormones was also determined for carbachol at 10^–3^ M and histamine at 10^–5^ M using the same experimental design used for LTB_4_. Neither DHT, E1, nor E2 incubated for 30 min blocked the carbachol-induced Ca^2+^ response in either males or females (Supplemental Data 3A,B,E). Similarly, neither DHT, E1, nor E2 incubated for 30 min blocked the histamine-induced Ca^2+^ response (Supplementary Fig. 3C,D,F).

**Figure 6:**
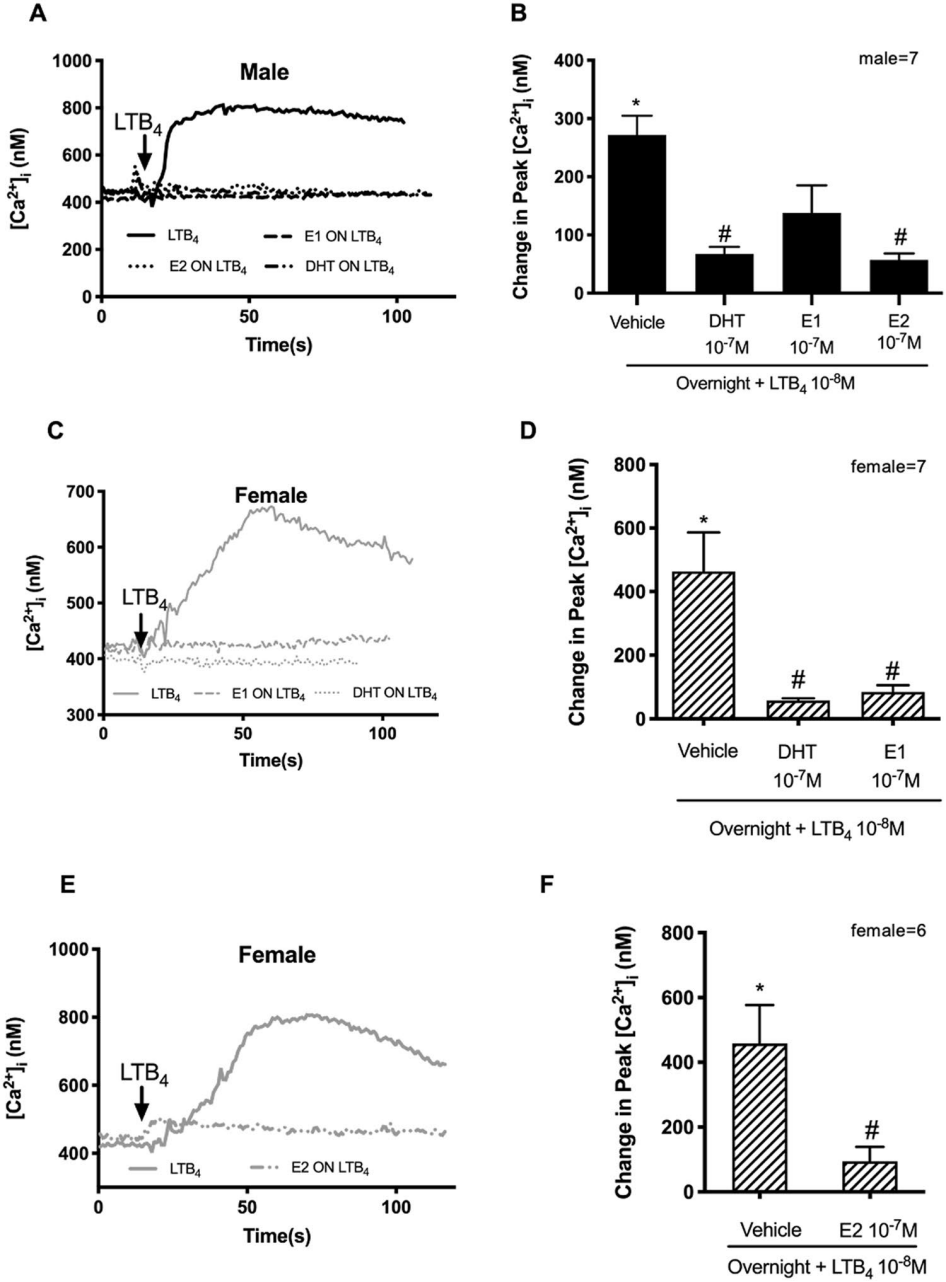
Overnight treatment with sex hormones inhibits the Ca^2+^ response from human conjunctival goblet cells cultured from male and female cells to the inflammatory mediator LTB_4_. Cultured goblet cells from male and female conjunctiva were treated with DHT, E1 or E2 (10^−7^ M) or vehicle overnight prior to addition LTB_4_ (10^−8^ M). [Ca^2+^]_i_ was measured by fura2 assay. The average [Ca^2+^]_i_ level over time was shown in (**A**, **C** and **E**); Change in peak [Ca^2+^]_i_ was calculated and shown in (**B**, **D** and **F**). Data are mean ± SEM. Number of individuals for each experiment are marked in graphs. Black lines and solid bars indicate data from male cells. Grey lines and slash-patterned bars indicate data from female cells. *Significance difference from basal. ^#^Significance difference between males and females.

To determine the action of long-term stimulation with sex hormones, goblet cells were incubated with DHT, E1, E2 or buffer for 24 h before adding LTB_4_. In male cells, LTB_4_ 10^–8^ M significantly increased the [Ca^2+^]_i_ to 404.70 ± 135.90 nM from basal (Fig. 6A,B). Treatment for 24 h with DHT or E2 significantly decreased the [Ca^2+^]_i_ to 63.21 ± 11.24 nM (*p* < 0.05); and 55.78 ± 9.49 nM (*p* < 0.05), respectively. In contrast, 24 h treatment with E1 dampened but did not significantly decrease the [Ca^2+^]_i_ to 127.60 ± 41.24 nM (Fig. 6B). In cells from females, LTB_4_ 10^–8^ M significantly increased the [Ca^2+^]_i_ to 463.90 ± 122.60 nM (Fig. 6C,D) and to 458.6 ± 118.3 (Fig. 6E,F) from basal. Incubation for 24 h with DHT, E1, or E2 each significantly decreased the LTB_4_-triggered [Ca^2+^]_i_ increase to 58.52 ± 6.54 nM (*p* < 0.05), 85.18 ± 21.16 nM (*p* < 0.05) (Fig. 6C,D), and 94.17 ± 44.96 nM (*p* < 0.05) (Fig. 6E,F), respectively.

The long-term action of sex hormones was determined for carbachol and histamine using the same experimental protocol as for LTB_4_. Neither DHT, E1, nor E2 incubated for 24 h blocked the carbachol- or histamine-induced Ca^2+^ response in either males or females (Supplemental Data 4).

In summary, sex hormones incubated for both the short-term and long-term inhibited the cellular Ca^2+^ response induced by the eicosanoid inflammatory mediator LTB_4_, but not the cholinergic agonist carbachol nor the autacoid histamine.

### Differential response of goblet cells from male compared to female conjunctiva to LXA_4_, but not RvD1 or RvD2

**Figure 7:**
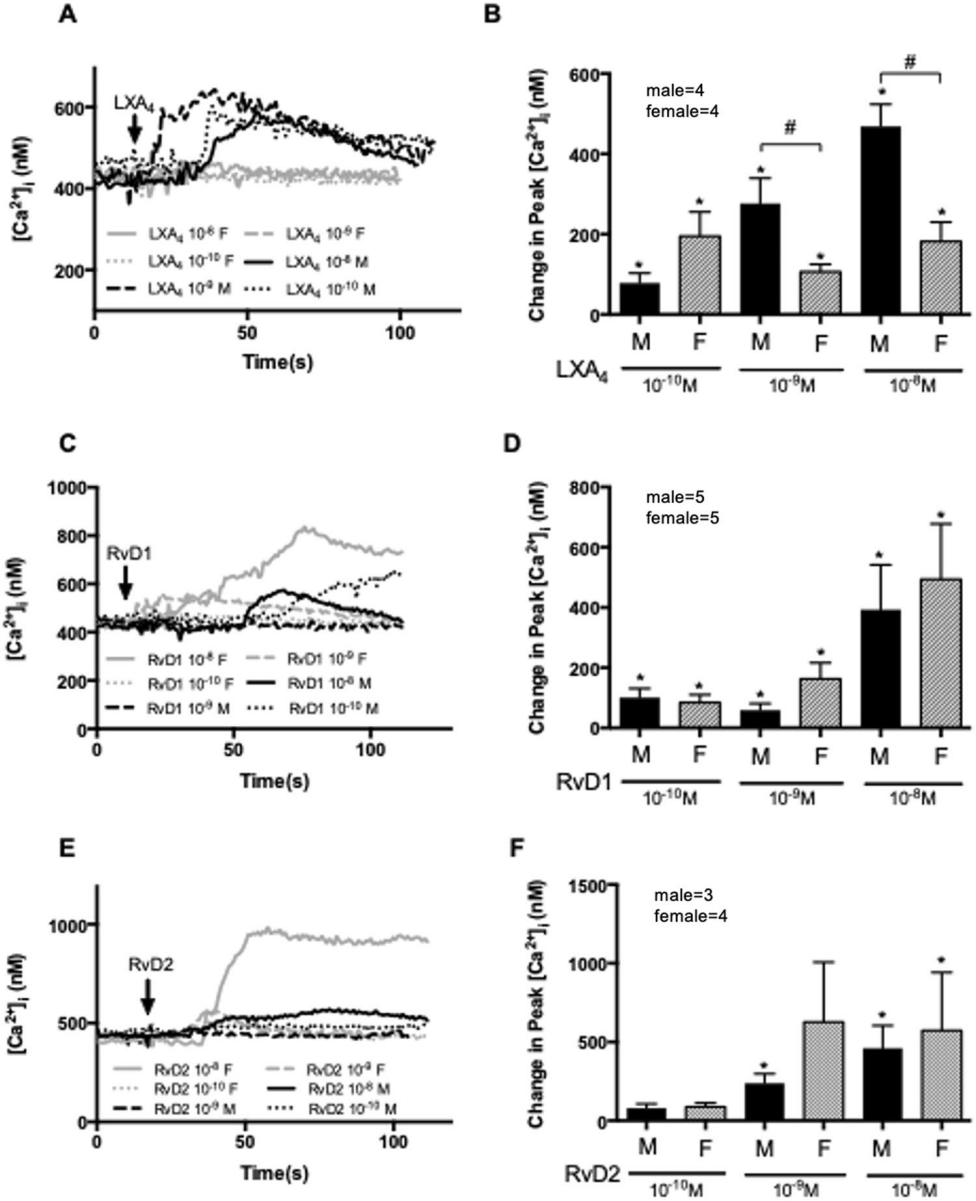
Cultured conjunctival goblet cell Ca^2+^ response to LXA_4_ from male is greater than female cells. Goblet cells from both sexes were stimulated with LXA_4_ 10^–10^–10^−8^ M (**A** and **B**) RvD1 10^–10^–10^−8^ M (C and D) or RvD2 10^–10^–10^−8^ M (**E** and **F**). [Ca^2+^]_i_ was measured by fura2 assay. The average [Ca^2+^]_i_ level over time was shown in (**A**, **C** and **E**). Change in peak [Ca^2+^]_i_ was calculated and shown in (**B**, **D** and **F**). Data are mean ± SEM, the number of individuals is marked in each graph. Arrow indicates the addition of stimuli. Black lines and solid bars indicate data from male cells. Grey lines and slash-patterned bars and indicate data from female cells. *Significance difference from basal. ^#^Significance difference between males and females.

We next determined the [Ca^2+^]_i_ response of goblet cells to the specialized pro-resolving mediators LXA_4_, RvD1, and RvD2. LXA_4_ is produced earlier in the resolution response than the resolvins as it is biosynthesized from arachidonic acid compared to DHA for the D-series resolvins^23^. When goblet cells cultured from male and female conjunctiva were stimulated with LXA_4_ (10^–10^–10^–8^ M) a concentration dependent-response was obtained in male but not female cells (Fig. 7A,B). When peak [Ca^2+^]_i_ was determined, a maximum response was obtained with LXA_4_ used at 10^–8^ M. This concentration significantly increased [Ca^2+^]_i_ to 468.6 ± 55.25 nM (*p* < 0.05). In contrast, in female cells, a maximum response was obtained with LXA_4_ used at 10^–10^ M. This concentration significantly increased [Ca^2+^]_i_ to 195.4 ± 60.57 (p < 0.05) from basal (Fig. 7A). The change in peak [Ca^2+^]_i_ triggered by 10^–9^ M and 10^–8^ M in males was significantly higher than females (*p* < 0.05), implying a significant sex-based difference in Ca^2+^ response to LXA_4_ (Fig. 7A,B).

RvD1 (10^–10^–10^–8^ M) caused a concentration dependent-response in male and female cells (Fig. 7C,D). The change in peak [Ca^2+^]_i_ in response to RvD1 was maximum at 10^–8^ M for cells from both sexes and was significantly increased to 393.1 ± 148.6 nM in male and 493.7 ± 183.9 nM in female cells (Fig. 7C,D). No significant difference was found when comparing the response from male and female cells stimulated with RvD1 at any concentration used (Fig. 7C,D). Similar results were obtained with RvD2 (10^–10^–10^–8^ M). RvD2-induced [Ca^2+^]_i_ increase was concentration-dependent and maximum at 10^–8^ M at which concentration the peak [Ca^2+^]_i_ was significantly increased to 573.5 ± 386.4 nM in males and 460.8 ± 144.1 nM in females (Fig. 7E,F). No significant sex-based differences were observed in the Ca^2+^ response to RvD2 at any concentration used.

In summary, human GCs cultured from male or female conjunctiva responded differently to LXA_4_, but no difference was found with the response to another family of SPMs, the D-series.

## Discussion

Several tear-producing ocular adnexa, including the lacrimal gland and the Meibomian glands, display sex-based differences in structure and function, resulting in sex-based differences in diseases such as dry eye and allergy^7–11^. Herein we show that message and location of protein for the receptors for estrogens are differently present in cultured human conjunctival goblet cells from both males and females. No AR protein was detected in female samples even though the mRNA was detected, a possible explanation of this finding is the prolonged lack of testosterone in culture. ARs in females are more dependent on the testosterone-induction, and the same action could have occurred with ERβ in male samples. The presence of ERα immunoreactivity in the nucleus in females suggests that ERα could bind to DNA to regulate the expression of target genes. Although the presence of the three sex hormone receptors in biopsy specimens from both sexes has been reported by other studies^28–30^, our results indicate there are differences with the expression of sex hormone receptors with the absence of sex hormones.

Even though the sex hormones themselves when tested did not alter the [Ca^2+^]_i_ in cells from either sex, they did affect the response to LTB_4_ and to the same extent in both males and females. Sex hormones have a variety of effects on cell function. The protective role of the androgen DHT has been observed^31,32^, and is consistent with the present study in which DHT decreased the action of the inflammatory mediator LTB_4_. Interestingly, we found that estrogen also attenuates the action of LTB_4_. The role of estrogen is controversial as reports demonstrate protective^33,34^, disruptive^35,36^, and neutral^37^ effects of estrogen in ocular surface diseases. Estrogen also has multiple types of actions. Estrogen regulates expression of a variety of genes involved in dry eye^38^. In addition, estrogen has more rapid non-gene expression action on cells, such as regulation of vascular endothelium by activating mitogen-activated protein (MAP) kinase kinase^39^. On the ocular surface, estradiol downregulates cyclic AMP (cAMP) signaling in human meibomian gland epithelial cells^3^. As the inflammatory mediator LTB_4_ activates the BLT1 receptor and is coupled to G_i_, the BLT1 receptor could work through cAMP in goblet cells. Furthermore, the BLT1 receptor also carries phosphorylation site for MAPK according to Scansite 4.0 https://scansite4.mit.edu/4.0/#home, indicating that it is possible for estrogen to modulate the activity of LTB_4_ through the BLT1 receptor. In summary, as one major consequence of goblet cell activation is mucin secretion, the results of our study indicate that sex hormones may protect the ocular surface from the excess tears and itchiness caused by mucin over-secretion during inflammation.

Sex hormones also demonstrate a long-term effect on the LTB_4_-Ca^2+^ response, but not on the cholinergic or histaminergic activated Ca^2+^ response. As sex hormones have receptors in the cell nuclei, they could affect the response through the synthesis of proteins. How sex hormones affect the expression of the receptors of the stimuli of each category needs to be further investigated.

We also investigated if neural, histaminergic, eicosanoid, and pro-resolution mediators have differential effects on the Ca^2+^ response in male compared to female human conjunctival goblet cells. We initially hypothesized that the unequal prevalence of conjunctival inflammatory diseases in men and women was due to different cellular responses to allergens or pathogens. However, the results of our experiments indicate that goblet cells from males and females are equally susceptible to histamine and LTB_4_ when sex hormones are eliminated from the culture conditions. Carbachol, a parasympathomimetic agonist, has an increased response in females compared to male goblet cells. These results are in contrast to the effect of sex hormones on the cholinergic agonist and LTB_4_, but not the histamine, responses. Cholinergic agonists have a sex-based difference in their action, but sex hormones do not affect this response. LTB_4_ stimulation does not have a sex-based difference in cutured goblet cells without sex hormones, but the sex hormones strikingly decrease the LTB_4_ response both short-term and long-term in cells from both sexes. These results suggest that very different cellular signaling mechanisms regulate sex based differences in agonist stimulated response compared to the effect of exogenously added sex hormones on these responses.

The distinct mechanisms facilitate sex-based differences in conjunctival goblet cells was also observed among pro-resolving mediators. When cultured goblet cells were activated with SPMs, there was a difference between goblet cells from males and females in their response to LXA_4_ only, with a higher Ca^2+^ response in males compared to females, among the three SPMs tested. LXA_4_ is one of the earliest discovered SPMs. Together with its receptor ALX/FPR2, LXA_4_ constitutes an essential protective eicosanoid circuit, regarded as the “jump start” of the resolution of inflammation^40^. LXA_4_ can be produced by several ocular tissues/cells, including the neutrophils in the corneal limbus, lacrimal glands, and cervical lymph nodes^13^, as well as the corneal epithelial cells^41^, and is present in the tears of healthy individuals^17^. The beneficial role of LXA_4_ in reducing chronic inflammation occurs by inhibiting the regulatory T cells in draining lymph nodes, which drive the inflammation towards chronicity^42^. Interestingly, a substantial decrease in lymph node LXA_4_ biosynthesis was found in an animal model of dry eye specifically in females rather than males^13^. An additional sex-based difference in LXA_4_ was found when estrogen was demonstrated to down-regulate the LXA_4_ circuit to induce delayed female-specific wound healing in the cornea^12^. Our current findings add new aspects of the relationship between LXA_4_ and sex, that is, sex also determines the cellular response to LXA_4_ in the epithelial conjunctival goblet cells. As LXA_4_ is biosynthesized from the same precursor as the pro-inflammatory mediator LTB_4_ and appears at an early stage of resolution of inflammation, we speculate that the active resolution of inflammation begins earlier in males than in females. This finding is consistent with increased disease found in female cells. The sex-based difference in the action of LXA_4_ on goblet cells also suggests that in future studies oo LXA_4_, attention should be paid to the sex differences in tissues, as analysis of both sexes together could cover up sex-based differences.

The D series resolvins such as RvD1 and RvD2 used in the present study are biosynthesized from DHA, a process that occurs later in resolution of inflammation than biosynthesis of LXA_4_. RvD1 and RvD2 have pro-resolution activities as does LXA_4_ and these D-series resolvins have protective effects on the ocular surface^20,21,43^. In the present study, however, we did not observe any sex-based differences in goblet cell responses to either RvD1 or RvD2. In many cell types RvD1 and LXA_4_ can use the same receptor, however, in human conjunctival goblet cells RvD1 and LXA_4_ use different receptors: RvD1 uses GPR32 and LXA_4_ uses ALX/FPR2^44^. RvD2 uses the GPR18 receptor (unpublished work by Botten, et al.). This could explain the different results for RvD1 and RvD2 compared to LXA_4_ obtained in the present study. In healthy human tears, we found a significantly higher level of RvD1 and RvD2 in males^17^. Evidence in mice peritonitis model showed elevated key enzymes in the SPMs synthesis, including 12/15-lipoxygenase in males with elimination of sex hormones by gonadectomy, but not in females ^45^. Thus we suggest that the sex based differences of LXA_4_ are mainly in the cellular response to LXA_4_, while those of RvD1/D2 are mainly the amount of biosynthesis by local tissues. The mechanisms behind the sex-based difference in the amount of biosynthesis of RvD1/D2 needs further investigation.

In conclusion, there are specific mediators that are altered in male versus female conjunctival goblet cell Ca^2+^ responses and whose Ca^2+^ response is affected by exogenous sex hormones using the androgen and estrogen receptors present in goblet cells from both sexes. There is, however, no widespread or generalized effect of sex hormones on goblet cell function. The major effect of the sex hormones is on proinflammatory mediators; the major sex-based difference is in the cellular response to the pro-resolution mediator LXA_4_. That males have higher response to the ω-6-fatty acid derived SPM LXA_4_ indicates that males may terminate inflammation in conjunctival goblet cells faster than females. Sex based differences should be taken into consideration when studying inflammation and its resolution in conjunctival inflammatory diseases.

## Methods

### Human materials

Human conjunctival tissue was obtained from Eversight Eye Bank (Ann Arbor, MI). All tissue was donated to the eye bank with the prior informed consent and authorization of the donors for use in scientific research. The tissues were placed in Optisol media within 6 h of death. Use of this tissue was reviewed by the Massachusetts Eye and Ear Human Studies Committee and determined to be exempt and does not meet the definition of research with human subjects. The age and sex of each individual donor are listed in Supplementary Table 1.

### Goblet cell culture

Goblet cells from human conjunctiva were grown in organ culture. Pieces of minced conjunctival epithelium were placed on culture dishes with RPMI 1640 medium supplemented with 10% fetal bovine serum (FBS), 2 mM glutamine, 2 mM non-essential amino acids (NEAA), 2 mM sodium pyruvate, and 100 μg/ml penicillin–streptomycin. The tissue pieces were removed after nodules of cells were observed. Twenty-four hours before experiments, cells were transferred into FBS-free and phenol red-free media to minimize the potential effect of sex hormones contained in FBS. First passage goblet cells were used in all experiments. Identity of cultured cells was periodically checked by evaluating staining with antibody to cytokeratin 7 (detects goblet cell bodies) and the lectin Helix pomatia agglutinin (HPA) (detects goblet cell secretory product) to ensure that goblet cells predominated.

### Immunofluorescence microscopy (IF)

First passage goblet cells were grown on glass coverslips and fixed with paraformaldehyde. The coverslips were blocked in 1% bovine serum albumin (BSA) with 0.2% Triton X-100 in PBS for 45 min. Anti-estrogen receptor (ER)α antibody, anti-ERβ antibody or anti-androgen receptor (AR) antibody (Abcam, Cambridge, UK) were used at a 1:100 dilution overnight at 4 °C. HPA conjugated to Alexa Fluor 488 (Invitrogen, Eugine, OR) was used at a 1:200 dilution. Secondary antibodies conjugated either to Cy2 or Cy3 (Jackson ImmunoResearch Laboratories, West Grove, PA) were used at 1:150 dilution for 1.5 h at room temperature. Negative control experiments included incubation with the absence of the primary antibody. Antibody specificity was determined by incubation with the primary antibody that was treated overnight with the corresponding immunizing peptide at a 1:5 ratio (Abcam, Cambridge, UK).

### Western Blotting Analysis (WB)

Cultured goblet cells were were homogenized in radio-immunoprecipitation assay buffer (RIPA, 10 mM Tris–HCl [pH 7.4], 150 mM NaCl, 1% deoxycholic acid, 1% Triton X-100, 0.1% sodium dodecyl sulfate, and 1 mM EDTA) in the presence of a protease inhibitor cocktail (Sigma-Aldrich, St Louis, MO). The homogenate was centrifuged at 2000*g* for 30 min at 4 °C. Proteins were separated by sodium dodecyl sulfate–polyacrylamide gel electrophoresis and processed for western blotting analysis. The same antibodies were used as in IF experiments, with 1:500 dilution. Immunoreactive bands were visualized by the enhanced chemiluminescence method. The specificity of the antibodies was confirmed using the corresponding peptide at a 1:5 ratio (Abcam, Cambridge, UK) as for IF.

### RT-PCR

Cultured goblet cells were homogenized in TRIzol (Invitrogen, Carlsbad, CA), and total RNA was isolated. The total RNA was purified using a TURBO DNA-free kit (Thermo Fisher, Waltham, MA). Five micrograms of purified total RNA were used for complementary DNA (cDNA) synthesis using the Superscript First-Strand Synthesis System for RT-PCR (Invitrogen, Carlsbad, CA). The cDNA was amplified by the polymerase chain reaction (PCR) using primers specific to human *Erα* and *Erβ* and *Ar* receptors using the Jumpstart REDTaq Readymix Reaction Mix (Sigma-Aldrich, St. Louis, MO) in a thermal cycler (Master Cycler, Eppendorf, Hauppauge, NY). The genome sequence was found in the NCBI database (https://www.ncbi.nlm.nih.gov) and primers were designed using primer blast (http://www.ncbi.nlm.nih.gov/tools/primer-blast) (*Erα* F: 5′-GGAGGGCAGGGGTGAA-3′ R: 5′-GGCCAGGCTGTTCTTCTTAG-3′; *Erβ* F: AGAGTCCCTGGTGTGAAGCAAG-3′ R: GACAGCGCAGAAGTGAGCATC-3′; *Ar* F: 5′-AATGGGACCTTGGATGGAGAACTA-3′ R: 5′-TCATAACATTTCCGGAGACGACAC-3′). The conditions were as follows: 5 min at 95 °C followed by 35 cycles of 1 min at 94 °C, 30 s at an annealing temperature of 62 °C for *Erα* and *Erβ*, 59 °C for *Ar*, and 1 min at 72 °C with a final hold at 72 °C for 10 min. Samples with no cDNA served as the negative control while the presence of *β-actin* was a positive control. Amplification products were separated by electrophoresis on a 1.5% agarose gel and visualized by ethidium bromide staining.

### Measurement of [Ca^2+^]_i_

First passage human conjunctival goblet cells were plated onto 35-mm glass-bottom culture dishes and incubated at 37 °C overnight. Cells were then incubated for 1 h at 37 °C with Krebs–Ringer bicarbonate buffer containing 119 mM NaCl, 4.8 mM KCl, 1.0 mM CaCl_2_, 1.2 mM MgSO_4,_ and 25 mM NaHCO_3_ with 4-(2-hydroxyethyl)-1- piperazineethanesulfonic acid (HEPES) plus 0.5% BSA containing 0.5 μM fura-2/AM (Invitrogen, Grand Island, NY, USA), 8 μM pluronic acid F127 (Sigma-Aldrich, St. Louis, MO, USA) and 250 μM sulfinpyrazone (Sigma-Aldrich) for 1 h. Before calcium measurements were started, cells were washed with KRB-HEPES containing sulfinpyrazone. Ca^2+^ measurements were conducted using a ratio imaging system (In Cyt Im2; Intracellular Imaging, Cincinnati, OH, USA) using wavelengths of 340 and 380 nm and an emission wavelength of 505 nm. Cells were stimulated with agonists, antagonists, and inhibitors. The intracellular [Ca^2+^] ([Ca^2+^]_i_) over time was recorded, and the change in peak [Ca^2+^]_i_ was calculated by subtracting the average of the basal value from the peak [Ca^2+^]_i_ value.

### Statistical analysis

The data are presented as the fold-increase above basal as average ± SEM. Student’s *t*-test was used in two group comparisons, and One-way ANOVA with Dunnett correction was used in multiple group comparisons. *P* < 0.05 was set as statistically significant.

### Ethical approval

Institutional Review Board Statement: The use of human tissue was reviewed by the Massachusetts Eye and Ear Human Studies Committee and determined to be exempt and does not meet the definition of research with human subjects.

## Supplementary Information

Supplementary Information.

## Data Availability

Raw data were generated at Schepens Eye Research Institute. Derived data supporting the findings of this study are available from the corresponding author D.A.D. on request.
