# Supplementary material for: Sex-based differences in conjunctival goblet cell responses to pro-inflammatory and pro-resolving mediators
Source: Sci Rep. 2022 Sep 29;12:16305. doi: 10.1038/s41598-022-20177-9 (PMC9522727; doi:10.1038/s41598-022-20177-9)
Supplement: Supplementary file 1 — Supplementary Information. [file 41598_2022_20177_MOESM1_ESM.pdf]

SUPPLEMENTARY DATA

Sex-Based Differences in Conjunctival Goblet Cell Responses to Pro-Inflammatory and Pro-Resolving Mediators

Menglu Yang, MD, PhD, Haakon K.Fjærvoll, Ketil A. Fjærvoll, Nicholas H. Wang , Tor P. Utheim, MD, PhD, Charles N. Serhan, PhD, DSc and Darlene A. Dartt, PhD

Supplementary Table 1: Sex and age of individual donors

NO.	SEX	AGE
**1**	M	76
**2**	M	70
**3**	M	65
**4**	M	64
**5**	M	48
**6**	M	68
**7**	F	56
**8**	F	64
**9**	M	74
**10**	F	71
**11**	M	58
**12**	F	75
**13**	M	57
**14**	M	70
**15**	F	71
**16**	M	58
**17**	F	64
**18**	F	61
**19**	F	48
**20**	F	55
**21**	F	55
**22**	F	74
**23**	M	73
**24**	M	54
**25**	M	71
**26**	M	75
**27**	M	63
**28**	F	46
**29**	M	67
**30**	M	52
**31**	M	72
**32**	F	57
**33**	M	57

Supp 1

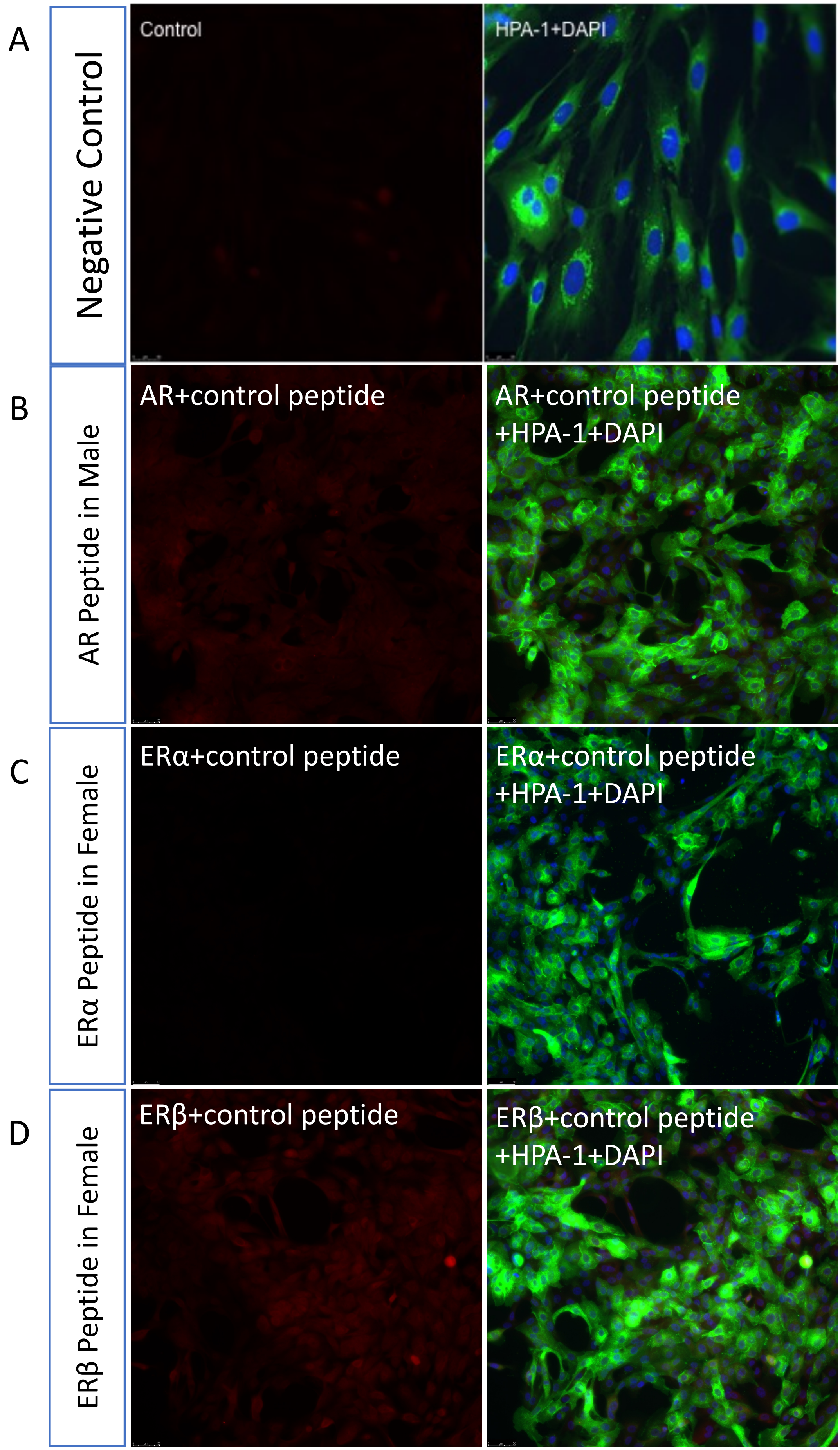
A
Negative Control
Control
HPA-1+DAPI
B
AR Peptide in Male
AR+control peptide
AR+control peptide
+HPA-1+DAPI
C
ERα Peptide in Female
ERα+control peptide
ERα+control peptide
+HPA-1+DAPI
D
ERβ Peptide in Female
ERβ+control peptide
ERβ+control peptide
+HPA-1+DAPI

Supplementary 1. Negative control and peptide control for ERα, ERβ, and AR staining in immunofluorescence microscopy. Incubation of isotype control antibody for IF is shown in (A). The overlay of control and HPA which binds to secretory products in goblet cells (green) and DAPI (blue) that binds to cell nuclei are shown on the right. IF images of AR, ERα, and ERβ after corresponding control peptide treatment are shown in (B, C and D). The overlay of control and HPA (green) and DAPI (blue) are shown on the right.

Supp 2

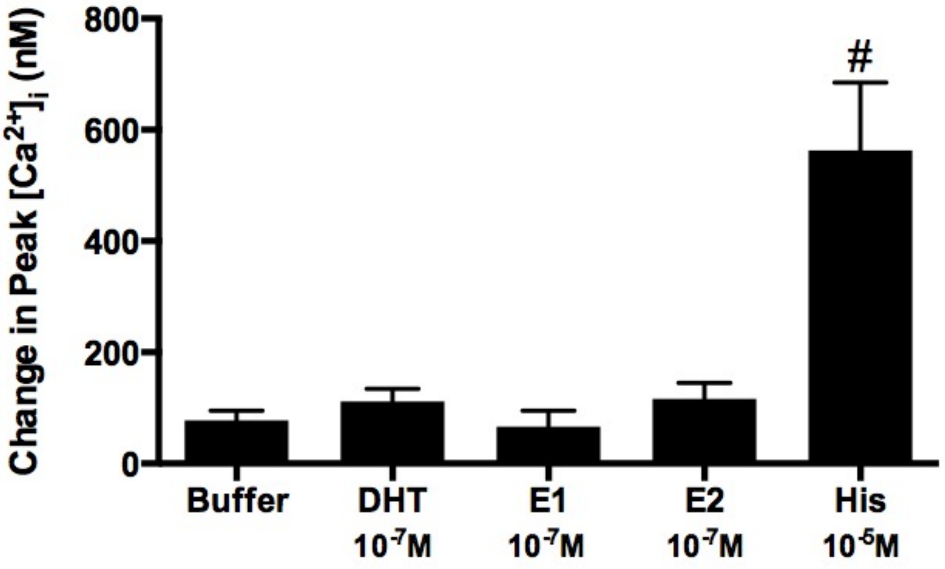

Supplementary 2: Addition of sex hormones alone did not increase $[Ca^{2+}]_i$ in cultured conjunctival goblet cells. DHT, E1 or E2 (10^{-7}M) was added to Fura 2 loaded conjunctival goblet cells. Vehicle (buffer) was added as negative control and histamine (10^{-5}M) was used as positive control. The increase of $[Ca^{2+}]_i$ was measured and the change in peak $[Ca^{2+}]_i$ is shown. Data are mean ± SEM from 3 individuals of mixed sexes. #Indicates significant difference from basal.

Supp 3

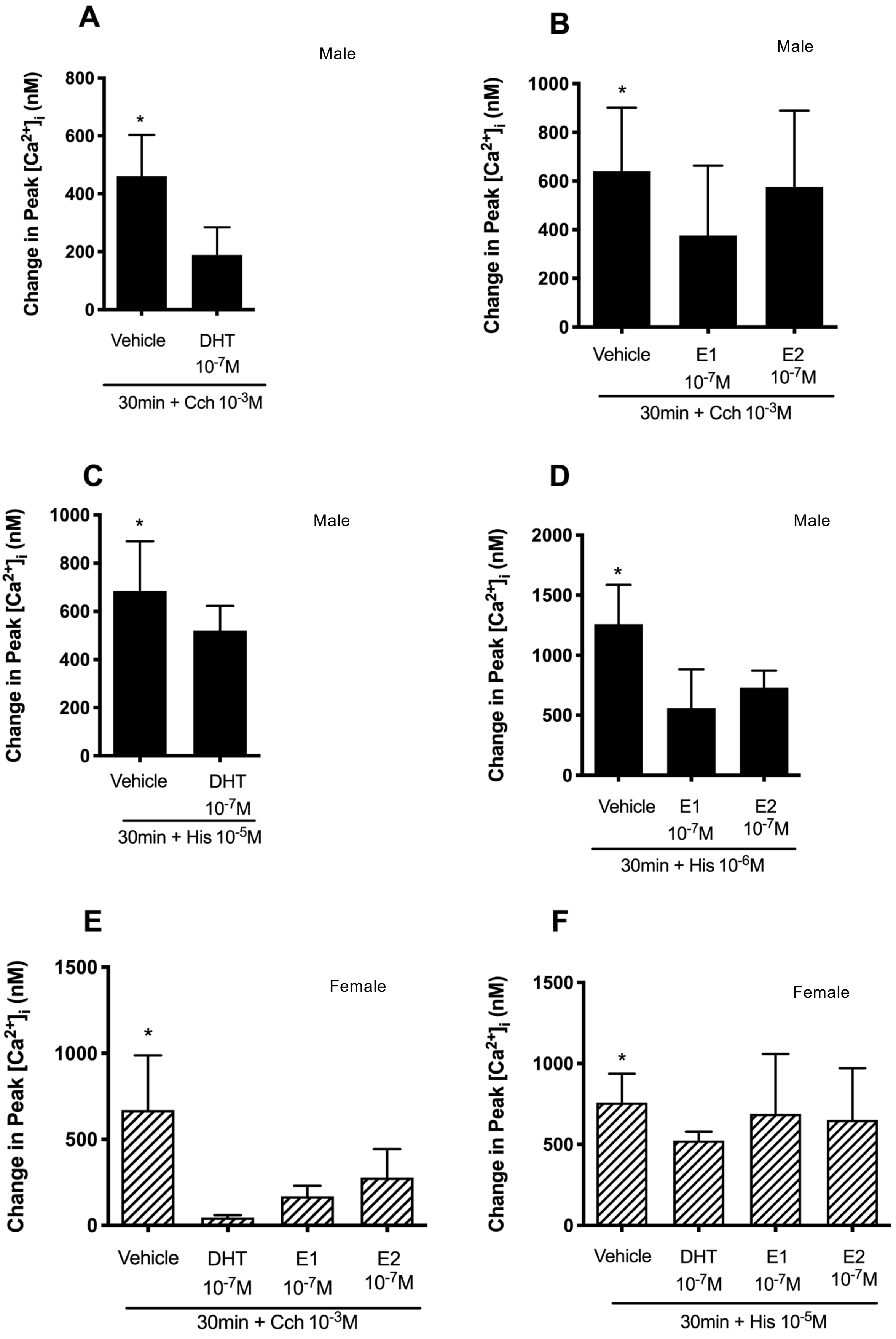
A
Male
Change in Peak [Ca2+]i (nM)
800
600
400
200
0
*
Vehicle
DHT 10-7M
30min + Cch 10-3M
B
Male
Change in Peak [Ca2+]i (nM)
1000
800
600
400
200
0
*
Vehicle
E1 10-7M
E2 10-7M
30min + Cch 10-3M
C
Male
Change in Peak [Ca2+]i (nM)
1000
800
600
400
200
0
*
Vehicle
DHT 10-7M
30min + His 10-5M
D
Male
Change in Peak [Ca2+]i (nM)
2000
1500
1000
500
0
*
Vehicle
E1 10-7M
E2 10-7M
30min + His 10-6M
E
Female
Change in Peak [Ca2+]i (nM)
1500
1000
500
0
*
Vehicle
DHT 10-7M
E1 10-7M
E2 10-7M
30min + Cch 10-3M
F
Female
Change in Peak [Ca2+]i (nM)
1500
1000
500
0
*
Vehicle
DHT 10-7M
E1 10-7M
E2 10-7M
30min + His 10-5M

Supplementary 3: Short-term treatment with sex hormones did not alter the male or female conjunctival goblet cell response to the cholinergic agonist (Cch) or the allergic mediator (histamine). Cultured goblet cells from or female cells were preincubated with DHT, E1 or E2(10^{-7} M), or vehicle for 30 min prior to addition of carbachol (Cch (10^{-3}M) or histamine (His, 10^{-6}M) and of Cch (10^{-3}M) (or His (10^{-6}M) $[Ca^{2+}]_i$ was measured by Fura2 assay. Data are mean ± SEM, n=8 for male and n=3 for female cells. Black lines indicate data from male cells, slash-patterned bars indicate data from females. *Indicates significant difference from basal.

Supp 4

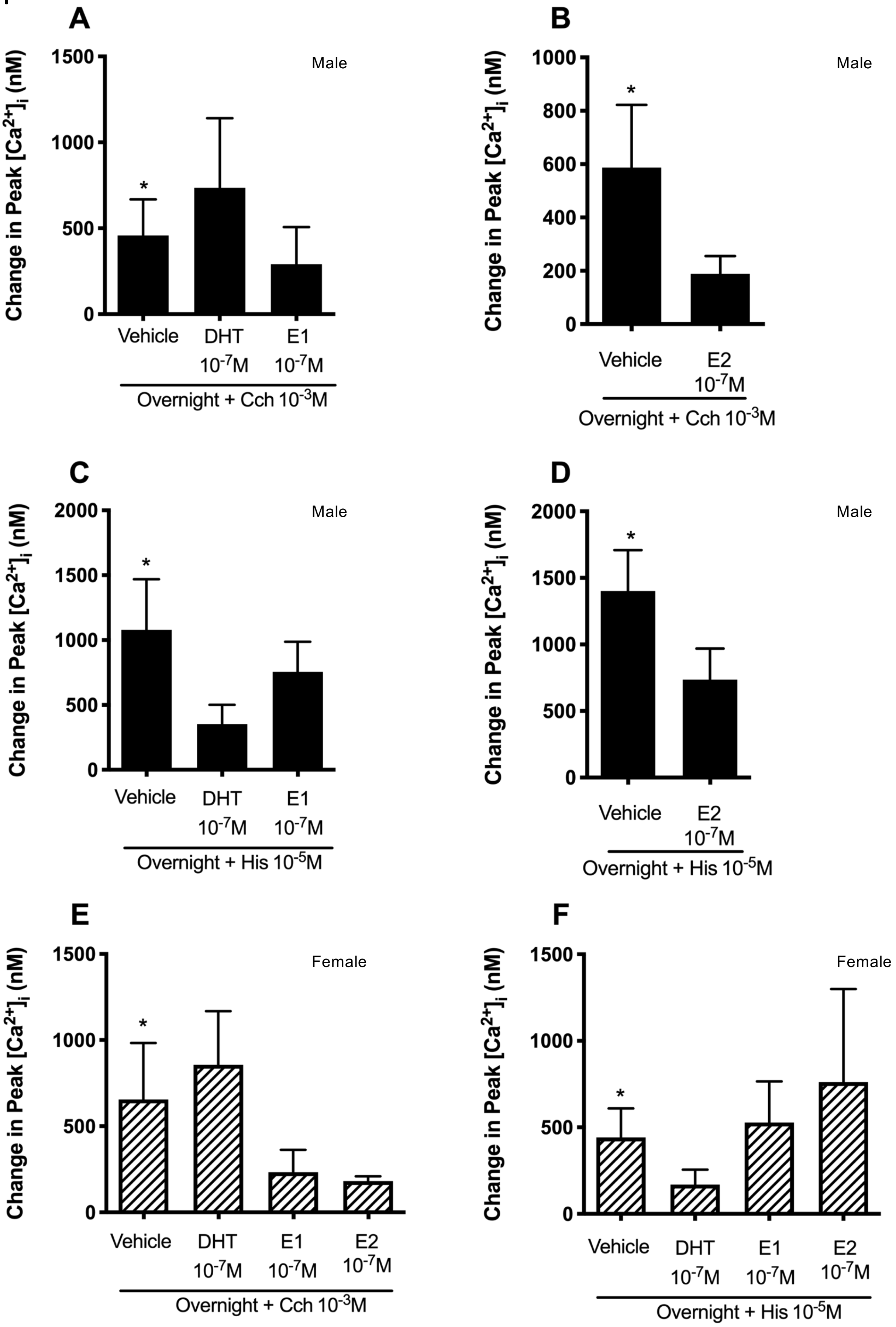
A
Male
Change in Peak [Ca2+]i (nM)
1500
1000
500
0
*
Vehicle
DHT 10-7M
E1 10-7M
Overnight + Cch 10-3M
B
Male
Change in Peak [Ca2+]i (nM)
1000
800
600
400
200
0
*
Vehicle
E2 10-7M
Overnight + Cch 10-3M
C
Male
Change in Peak [Ca2+]i (nM)
2000
1500
1000
500
0
*
Vehicle
DHT 10-7M
E1 10-7M
Overnight + His 10-5M
D
Male
Change in Peak [Ca2+]i (nM)
2000
1500
1000
500
0
*
Vehicle
E2 10-7M
Overnight + His 10-5M
E
Female
Change in Peak [Ca2+]i (nM)
1500
1000
500
0
*
Vehicle
DHT 10-7M
E1 10-7M
E2 10-7M
Overnight + Cch 10-3M
F
Female
Change in Peak [Ca2+]i (nM)
1500
1000
500
0
*
Vehicle
DHT 10-7M
E1 10-7M
E2 10-7M
Overnight + His 10-5M

Supplementary 4: Overnight treatment with sex hormones did not alter the male or female conjunctival goblet cell response to the cholinergic agonist carbachol (Cch) or the allergic mediator histamine (His). Cultured goblet cells from male and female cells were preincubated with DHT, E1 or E2(10^{-7} M) or vehicle for overnight prior to addition of Cch (10^{-3}M) or histamine (10^{-6}M). $[Ca^{2+}]_i$ was measured by Fura2 assay. Data are mean ± SEM, n=4 for male and n=3 for female cells. Solid bars indicate data from male cells, slash-patterned bars indicate data from female cells. *Indicates significance difference from basal.

Supplementary 5: Raw data for RT-PCR

Era

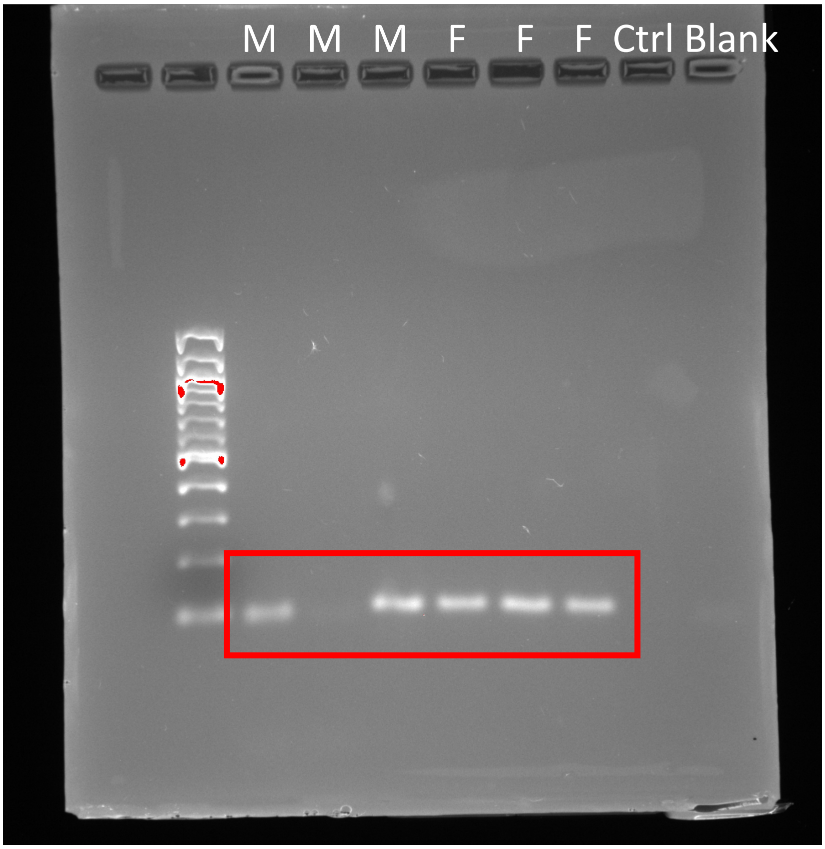

Raw image for Fig 1 A

Era

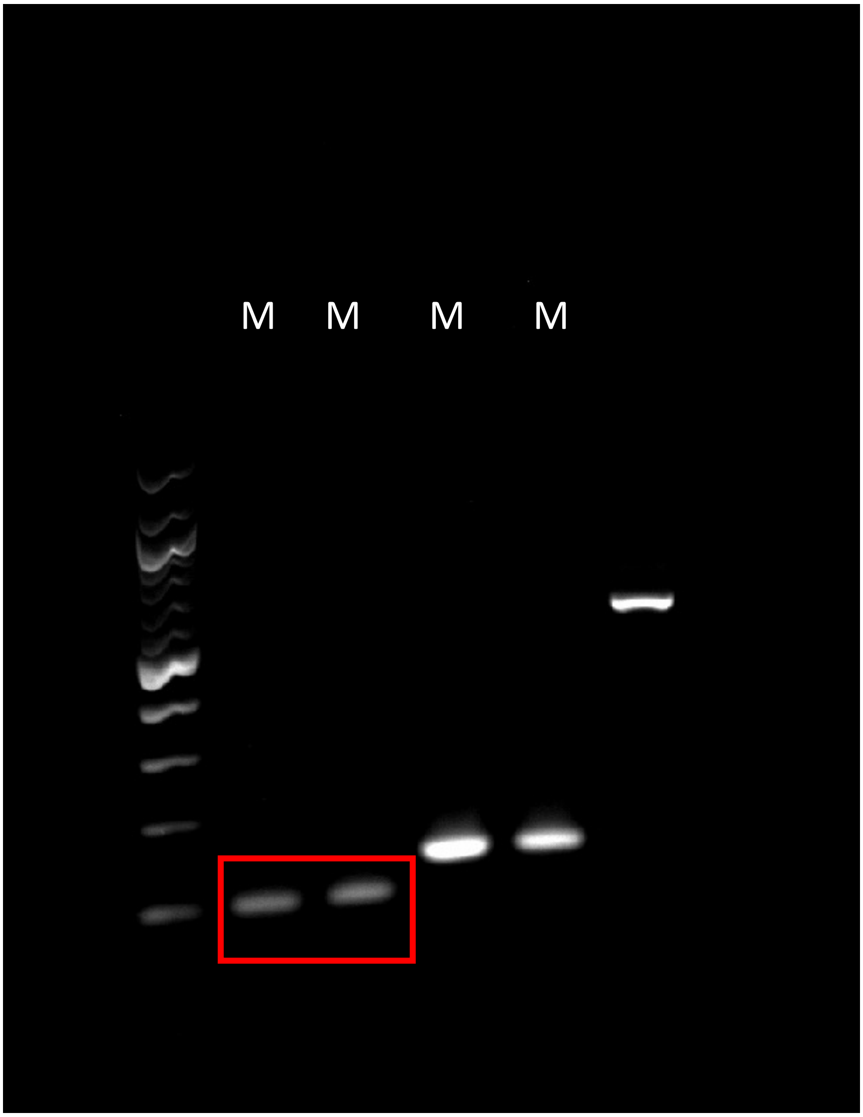

Raw image
for Fig 1 A
(1st and 2nd lane)

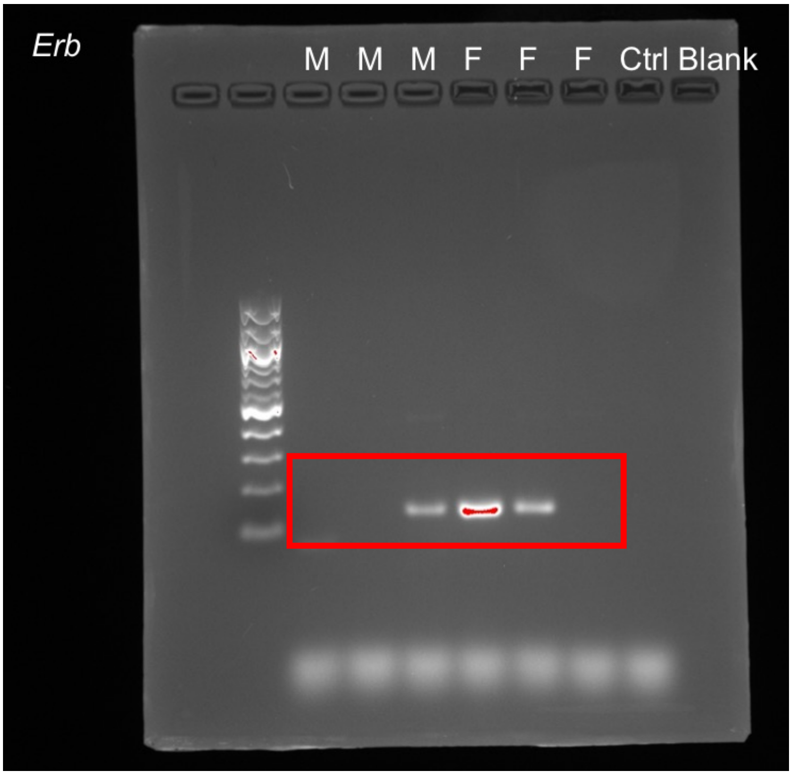

Raw image
for Fig 2 A

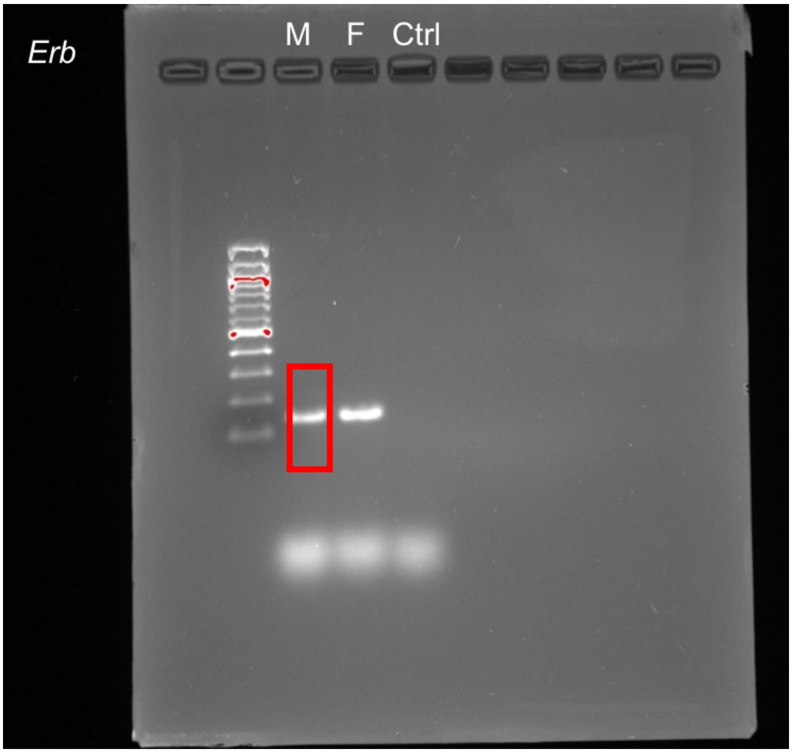

Raw image
for Fig 2 A
(F here act as
positive control)

Ar

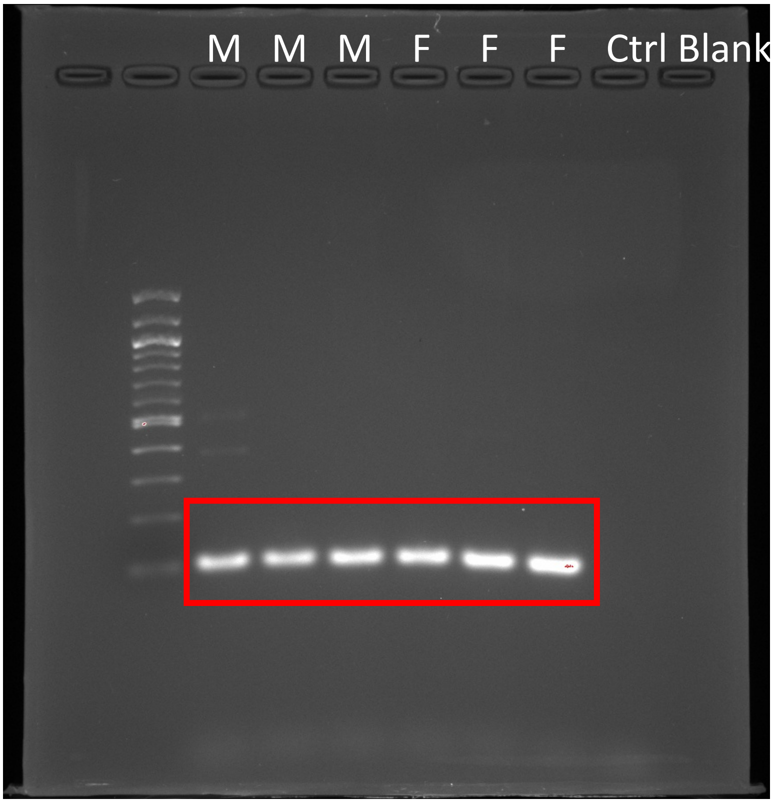

Raw image for Fig 3 A

Supp 6: Raw data of WB

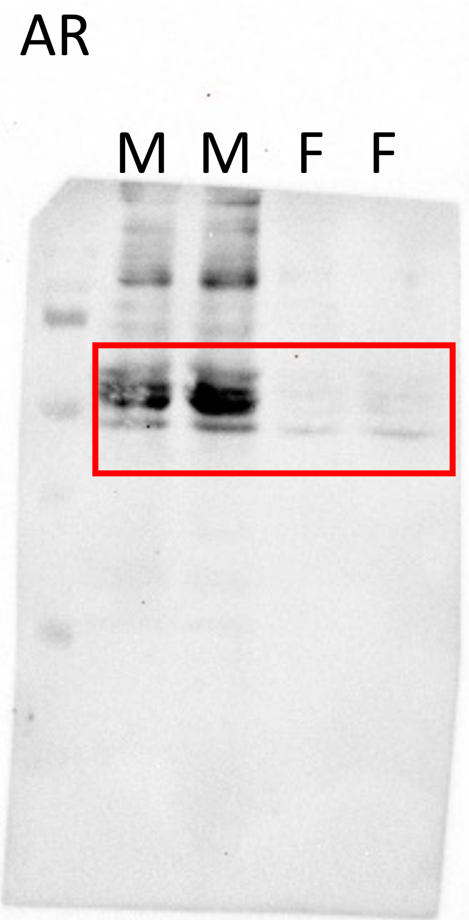

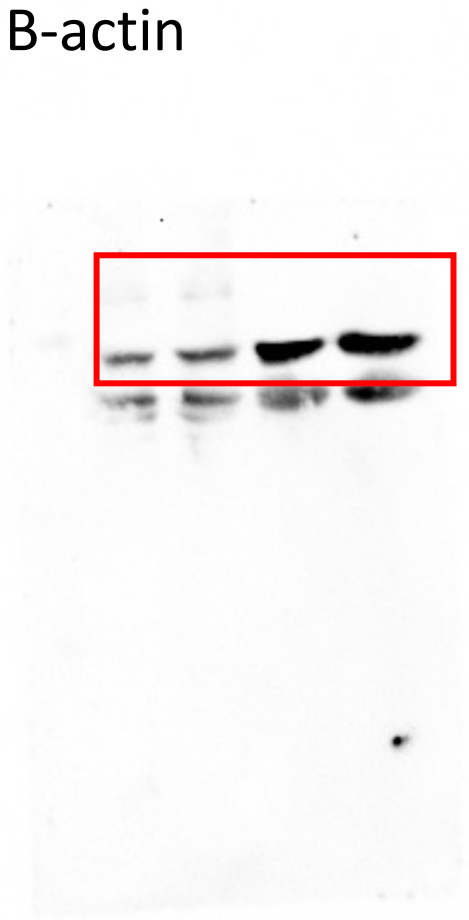

Raw data for Fig 3B (first 4 lines)

AR

B-actin

F F F

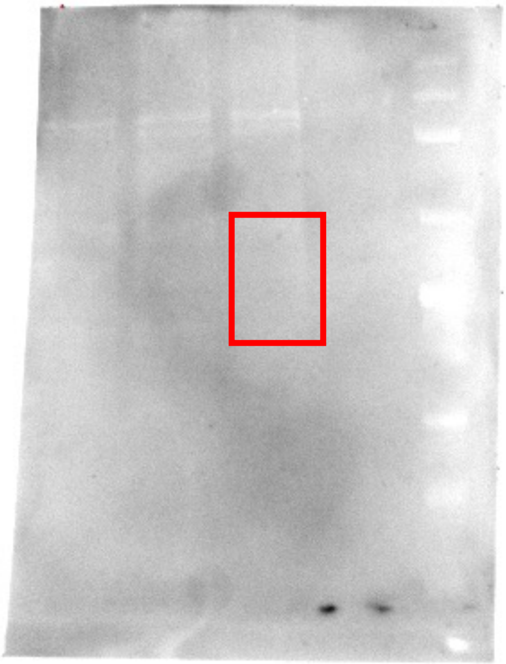

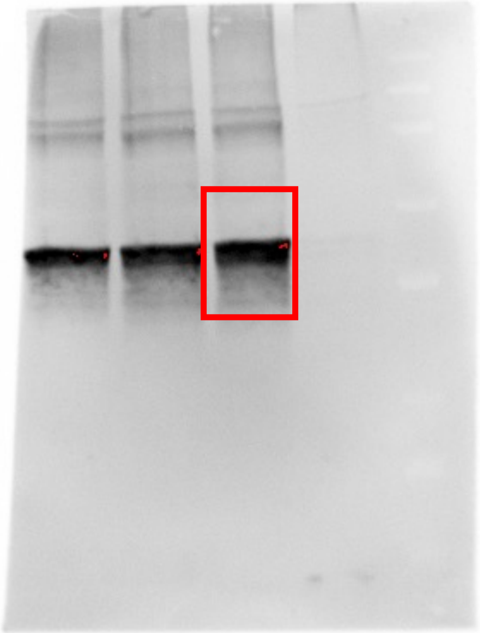

Raw data for Fig 3B (5th line)

ERa

M F F F M M

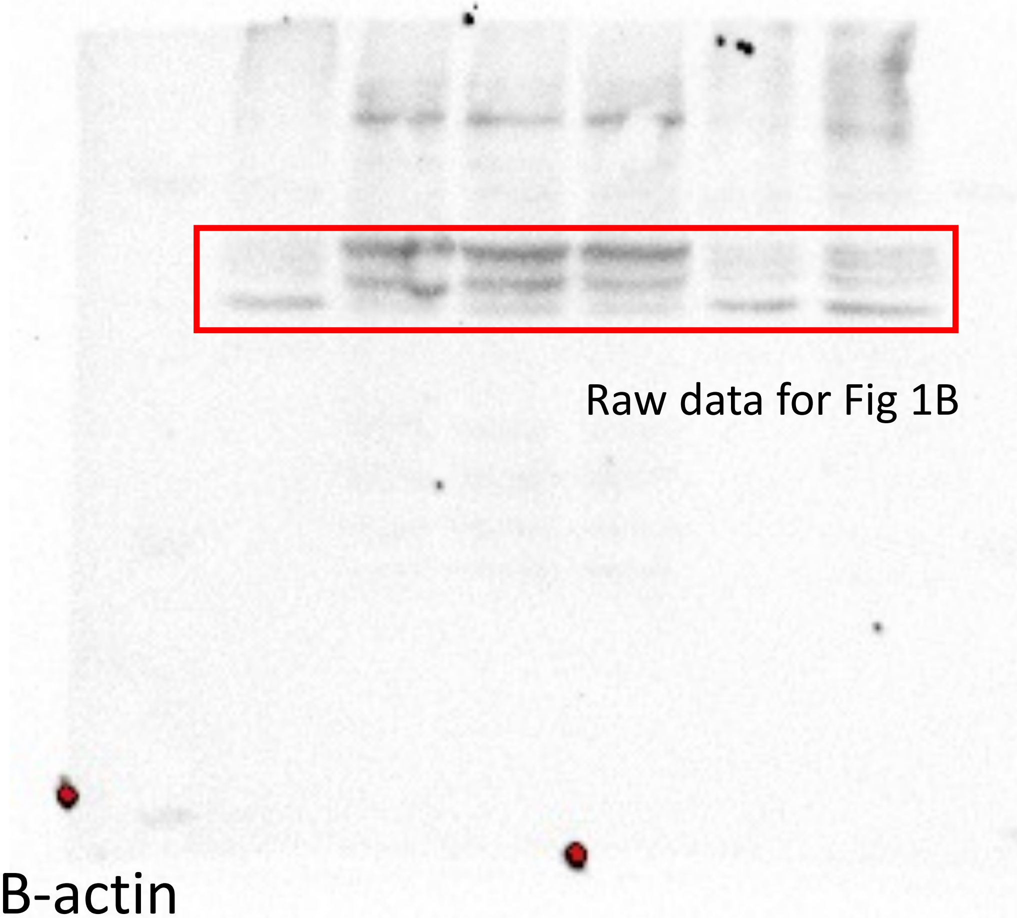

B-actin

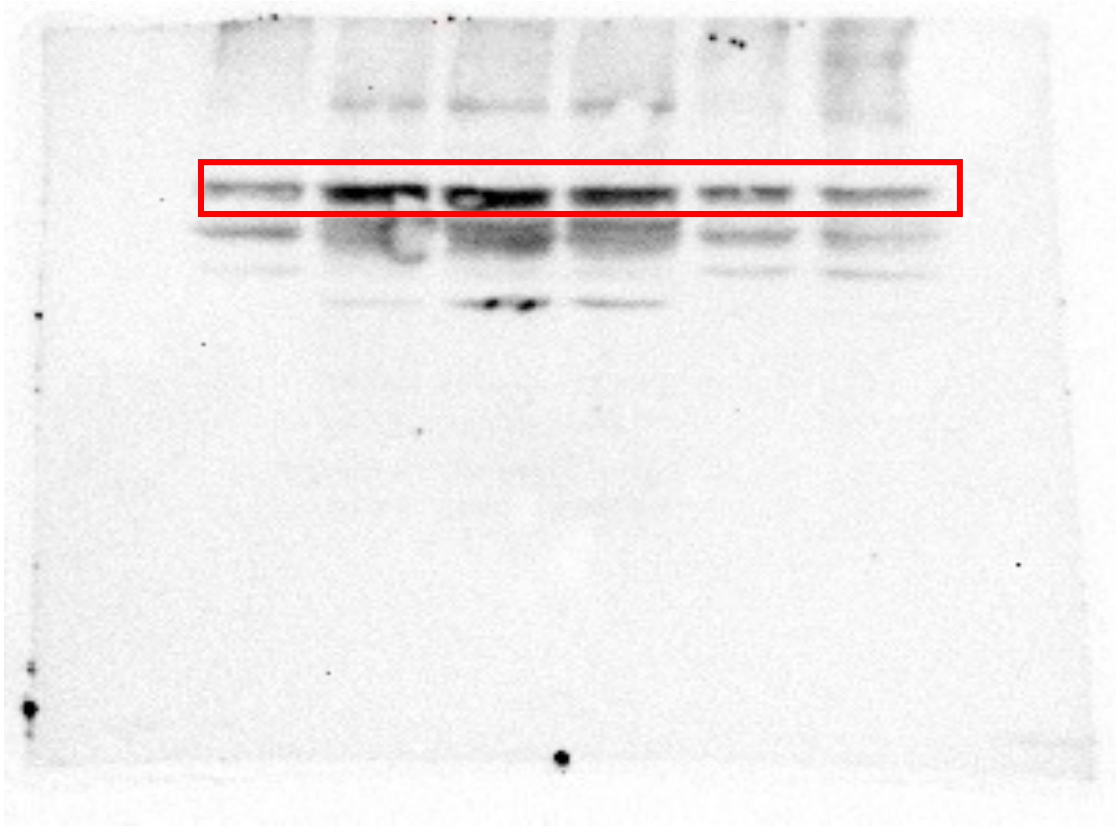

ERb

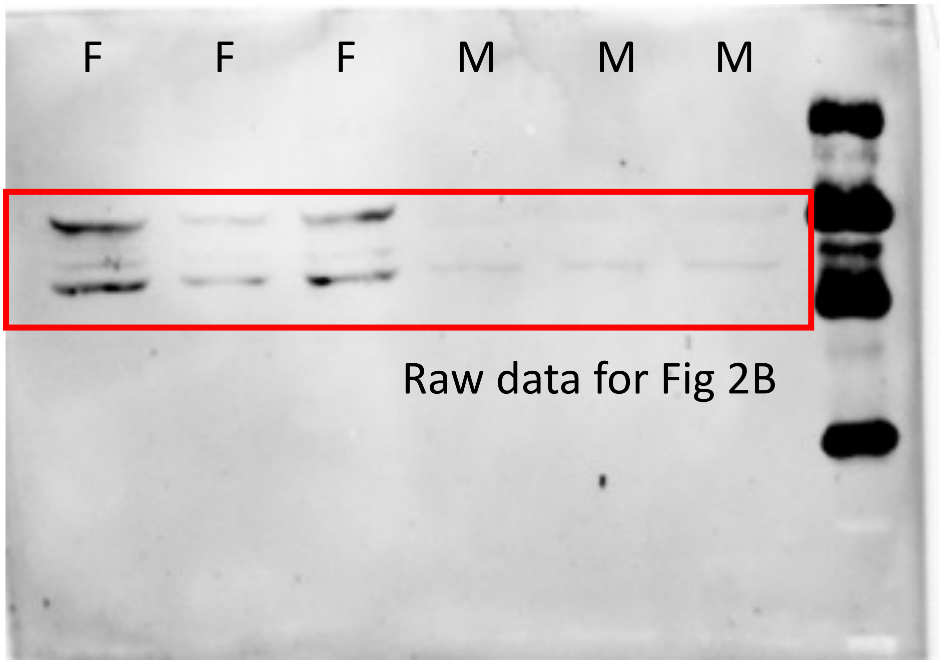

B-actin

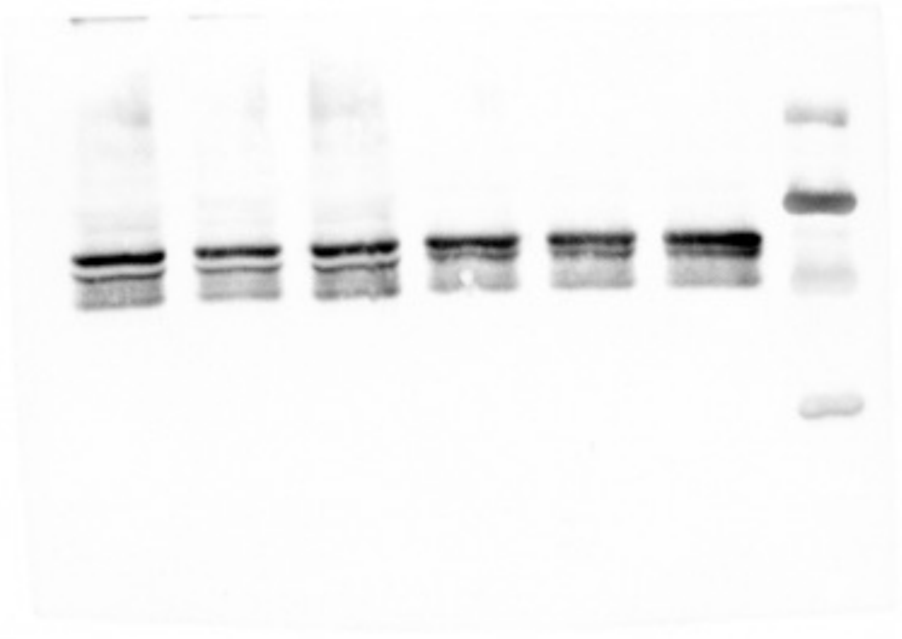

Supplementary 6: Raw data for WB

Supp 7

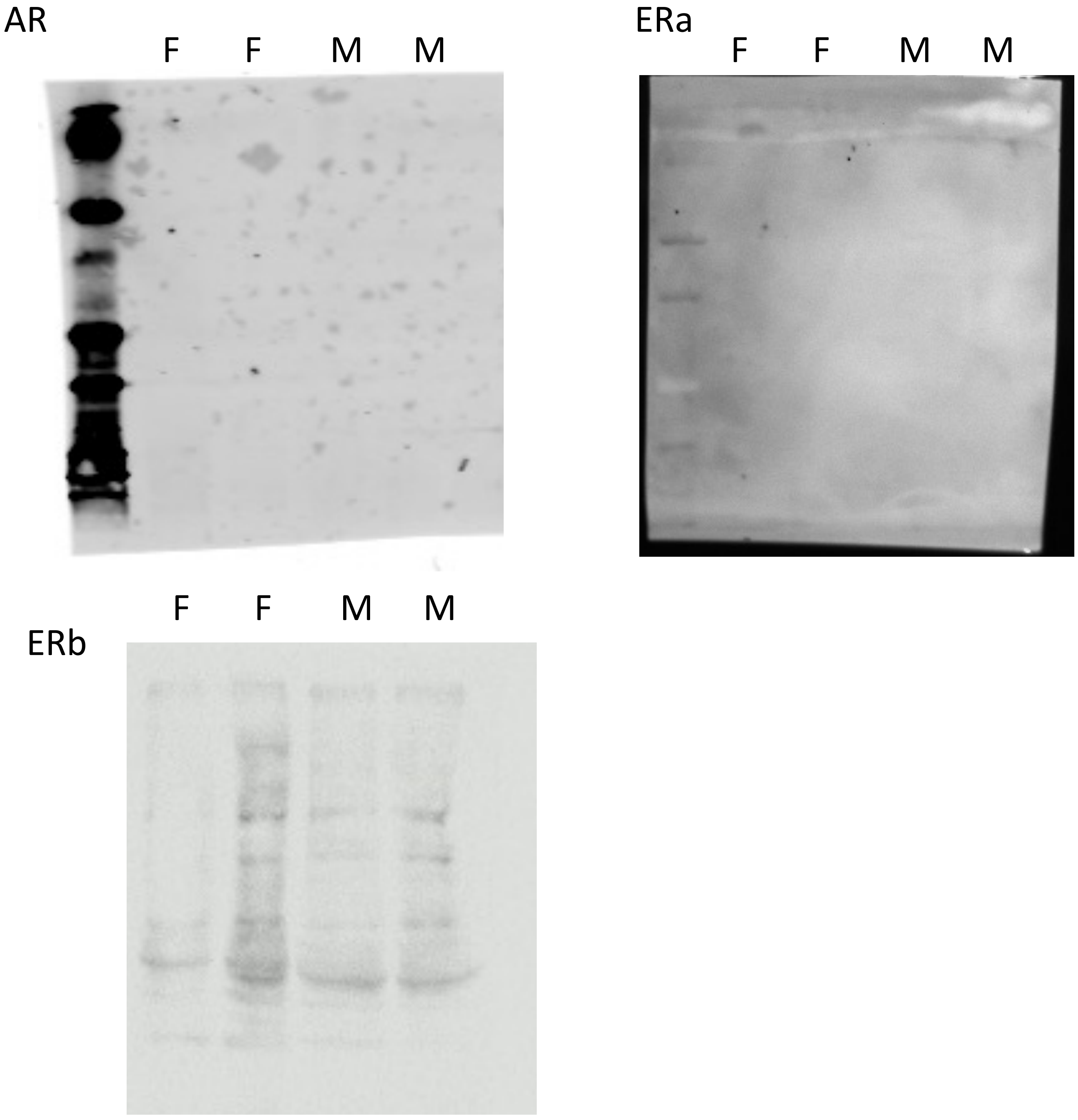

Supplementary 7: Peptide control for ERα, ERβ, and AR in WB.
